# Molecular networks affected by neonatal microbial colonization in porcine jejunum, luminally perfused with enterotoxigenic *Escherichia coli*, F4ac fimbria or *Lactobacillus amylovorus*

**DOI:** 10.1371/journal.pone.0202160

**Published:** 2018-08-30

**Authors:** Paolo Trevisi, Davide Priori, Alfons J. M. Jansman, Diana Luise, Sietse-Jan Koopmans, Ulla Hynönen, Airi Palva, Jan van der Meulen, Paolo Bosi

**Affiliations:** 1 DISTAL, University of Bologna, Bologna, Italy; 2 Wageningen UR Livestock Research, Wageningen, The Netherlands; 3 Department of Veterinary Biosciences, Division of Microbiology and Epidemiology, University of Helsinki, Helsinki, Finland; Evonik Industries AG, GERMANY

## Abstract

The development of an early complex gut microbiota may play an important role in the protection against intestinal dysbiosis later in life. The significance of the developed microbiota for gut barrier functionality upon interaction with pathogenic or beneficial bacteria is largely unknown. The transcriptome of differently perfused jejunal loops of 12 caesarian-derived pigs, neonatally associated with microbiota of different complexity, was studied. Piglets received pasteurized sow colostrum at birth (d0), a starter microbiota (*Lactobacillus amylovorus* (LAM), *Clostridium glycolicum*, and *Parabacteroides*) on d1-d3, and a placebo inoculant (simple association, SA) or an inoculant consisting of sow’s diluted feces (complex association, CA) on d3-d4. On d 26–37, jejunal loops were perfused for 8 h with either enterotoxigenic *Escherichia coli* F4 (ETEC), purified F4 fimbriae, LAM or saline control (CTRL). Gene expression of each intestinal loop was analyzed by Affymetrix Porcine Gene 1.1_ST array strips. Gene Set Enrichment Analysis was performed on expression values. Compared to CTRL, 184 and 74; 2 and 139; 2 and 48 gene sets, were up- and down-regulated by ETEC, F4 and LAM, respectively. ETEC up-regulated networks related to inflammatory and immune responses, RNA processing, and mitosis. There was a limited overlap in up-regulated gene sets between ETEC and F4 fimbriae. LAM down-regulated genes related to inflammatory and immune responses, as well as to cellular compound metabolism. In CA pigs, 57 gene sets were up-regulated by CA, while 73 were down-regulated compared to SA. CA up-regulated gene sets related to lymphocyte modulation and to cellular defense in all loop perfusions. In CA pigs, compared to SA pigs, genes for chemokine and cytokine activity and for response to external stimuli were down-regulated in ETEC-perfused loops and up-regulated in CTRL. The results highlight the importance of the nature of neonatal microbial colonization in the response to microbial stimuli later in life.

## Introduction

After early studies comparing germ-free animals with conventionally reared animals, there is a general consensus that the programmed maturation of the GIT of neonate and suckling mammals requires the initial qualitative and quantitative colonization by environmental bacteria [1].

The succession of bacteria in the developing gut microbial community apparently follows some host-species-specific directions [2–3], notwithstanding the presence of inter-individual variation. The initial acquisition and subsequent establishment of intestinal bacteria are assumed to be of particular relevance in developing appropriate innate immunity (physical barrier and secreted defense molecules, complement function, and specialized defense cells) upon recognition of bacterial components and microbial metabolites originating from fermentation of dietary and endogenous constituents. In addition, the adaptation of the acquired immune system, including the capacity to tolerate commensals, is generated by antigen recognition and regulated by memory cells [4].

It is still not clear, however, whether the presence of just a few microbial species is sufficient to have a beneficial effect on the development of the gut community or whether interactions with a complex microbiota are required. This is particularly relevant in case of external interventions, like the administration of single probiotic species or probiotic cocktails, to improve gastrointestinal health in early life.

In pigs it is urgent to identify early strategies to prevent dysbiosis and to reduce the susceptibility to disease. Laycook et al. [5] identified bacterial strains with a positive influence in early microbial colonization to develop ways to improve the intestinal barrier and immune responses. Such an additional protection would be particularly important during the critical post-weaning period, when the animal is exposed to several risk factors including maternal absence, change of the diet, animal mixing, transient anorexia, and breaching of the immune defenses.

Enterotoxigenic *Escherichia coli* (ETEC) expressing F4 fimbriae is one of the pathogens that most frequently takes advantage of the post-weaning stress in pigs, causing the development of colibacillosis. The diarrhoeic syndrome is caused by the diffusion of various toxins into the body generating a complex inflammatory cascade [6]. The F4 fimbriae are necessary for the adhesion of ETEC to jejunal villi and for the development of the pathology.

Among the commensal bacteria frequently isolated from pigs, *Lactobacillus amylovorus*, including strains previously identified as *Lactobacillus sobrius*, is particularly interesting because a) it is one of the potential favorable early strains in the gut of pigs proposed by Laycook et al. [5]; b) it has been associated with diets including fermentable dietary fiber and oligosaccharides, that in turn favored a more diverse and balanced gut microbiota [7]; c) it was able to prevent ETEC-induced membrane barrier damage in porcine intestinal IPEC-1 epithelial cells [8]) and d) it contrasted post-infection multiplication of ETEC in vivo [9].

It has been generally recognized that the intestinal mucosa has an ability to orchestrate adaptation upon the encounter of different bacteria. However, studying the interaction of an early priming colonization induced by different microbial populations requires complex models that take into account a large number of potential stimuli caused by both pathogenic and commensal bacteria, as well as effects of genotype-related variations.

The aim of the present study was to elucidate gene expression networks that characterize the individual responses after the perfusion of the small intestinal tissue with beneficial or pathogenic bacterial strains in pigs that were differentially associated either with a simple or complex microbiota during their first days of life.

## Materials and methods

Ethics statement: The protocol of the study was approved by the Committee on the Ethics of Animal Experiments of Wageningen University and Research Centre in Lelystad, The Netherlands (Permission Number: 2012083.e).

Piglets were obtained from sows [(Great York × Pietrain) × ‘Dalland’ cross] by caesarean delivery (**CD**) (day 0) and were divided over two microbiota association treatments, housed in separate clean, non-sterile rooms and balanced for body weight (BW) and litter of origin. Piglets were housed in two pens per room suited with an automatic feeding system for supply of a moist diet. At 1 h and 5 h after birth, each CD-piglet received 45 mL pasteurized (30 min at 60 °C) sow colostrum by oral gavage [10–11]. All piglets received a simplified starter microbiota consisting of *Lactobacillus amylovorus* (**LAM**) (3.6 × 10^7^ CFU), *Clostridium glycolicum* (5.7 × 10^7^ CFU), and *Parabacteroides* spp. (4.8 × 10^7^ CFU) by oral inoculation (2 mL) on days 1, 2, and 3 after birth. These species are the most frequently identified in the intestine of piglets among the phylogenetic groups, and were previously proposed as minimal intestinal colonization microbiota for gnotobiotic pigs [5]. On days 3 and 4 of age, the piglets received either a complex microbiota by a 2 mL oral inoculant consisting of 10% saline diluted feces of an adult sow (complex association, **CA**) or a placebo inoculant (10% saline) (simple association, **SA**). The average birth weight was 1273 ± 138 g and 1275 ± 153 g for CA and SA piglets, respectively. Piglets were fed a milk replacer diet ad libitum during a period of 5 d (days 0–4). It consisted of bovine skimmed milk powder, whey powder, vegetable oil, hydrolyzed wheat protein, wheat starch, sucrose and a vitamin and mineral premix, and contained 230 g crude protein per kg milk replacer. A moist diet based on whey powder, maize, wheat, toasted full fat soybeans, oat flakes, sucrose, soybean meal, vegetable oil, coconut oil, wheat gluten, potato protein, rice protein, and brewer’s yeast was fed during the rest of the study days.

On days 26–37 of age, intestinal loops were prepared using the in situ small intestinal segment perfusion (**SISP**) technique in 6 CA and in 6 SA piglets, obtained from the previous group of animals and as described by Nabuurs et al. [12] and Kiers et al. [13]. In brief, the abdomen was opened along the linea alba and four small intestinal segments of 20 cm in length each were made in the mid-jejunum, starting at a distance of 200 cm distal to the ligament of Treitz. These segments retained their vascularization and were cannulated with a tube at the proximal and distal ends to perfuse and collect fluid, respectively.

For each pig four different isolated jejunal loops were perfused with 8 mL fluid per h for 8 h (saline with 0.1% glucose and 0.1% mixture of free amino acids). In each loop the introduced perfusion fluids contained: a) saline as a control (**CTRL**); b) saline + ETEC as a pathogenic strain (5.5 x 10^9^ CFU) (**ETEC**); c) saline + F4 fimbriae (0.5 mg) (**F4)**; or d) saline + LAM (8.1 x 10^8^ CFU) (**LAM**).) During the procedure, the pigs remained apparently healthy. At the end of the perfusion study, the animals were deeply anaesthetized with sodium thiopenthal (10 mg/kg body weight) and sacrificed by an intracardiac injection of Tanax^®^ (Intervet Italia, Italy, 0.5 mL/kg body weight). One sample of jejunal tissue per loop was collected and snap-frozen in liquid nitrogen for further molecular analysis.

The F4 fimbriae were purified from the same ETEC strain with the procedure reported by Van den Broeck et al. [14]. The dose of F4 was the same as used in a previous intestinal perfusion study of Danabassis [15]. The quantity of F4 fimbriae supplied with the perfusion fluid is approximately equivalent to thirty times the amount of F4 fimbriae present on the ETEC introduced in ETEC -inoculated loops, considering a concentration of 1.7 mg F4 fimbriae per 10^12^ bacteria [14].

### RNA isolation and gene quantification by real-time RT-qPCR

Total RNA was isolated from the intestinal tissue samples using the FastPure RNA kit (TaKaRa Bio Inc., Shiga, Japan). All procedures were in agreement with the manufacturer’s protocols. RNA purity and integrity were evaluated by an Agilent Bioanalyzer 2100 (Agilent Technologies, Palo Alto, CA) just before real-time quantitative PCR (RT-qPCR) analysis.

Total RNA was hybridized on Affymetrix Porcine Gene 1.1 ST array strips. Hybridized arrays were scanned using a GeneAtlas imaging station (Affymetrix, Santa Clara, CA, USA). Performance quality tests of the arrays, including the labelling, hybridization, scanning and background signals by a Robust Multichip Analysis, were performed on the CEL files using an Affymetrix Expression Console. The intensity records were log2-transformed. Transcript data have been submitted to the National Center for Biotechnology Information's Gene Expression Omnibus (NCBI GEO) with the GEO accession number GSE77787.

The expression of the C-C motif chemokine ligand 5 (*CCL5*), glutathione peroxidase 2 (*GPX2*), interleukin 22 (*IL22*), regenerating family member 3γ (*REG3G*) and trefoil factor 3 (*TFF3*) genes was quantified by RT-qPCR. One microgram of total RNA was reverse-transcribed using the ImProm-II Reverse Transcription System (Promega, Milan, Italy). Primers were designed based on a specific porcine nucleic acid sequence (Gen-Bank database) using Primer 3 version 0.4.0 (http://frodo.wi.mit.edu/primer3/). The RT-qPCR reactions were performed in a LightCycler Real-Time PCR Systems instrument (Roche Applied Science, Basel, Switzerland) by a shuttle PCR (2 steps, denaturation and annealing/extension) following the procedure described by Trevisi et al. [16]. The raw expression values obtained for *REG3G* were normalized by geometric averaging of two internal control genes: *HMBS* and the TATA box binding protein (*TBP*), following Nygard et al. [17]. Primers and amplification conditions for the genes are reported in Table 1.

**Table 1:** Primers information and RT-qPCR conditions used in the trials.

Gene^a^	NCBI accession number	Oligo sequence (5'→3'): Forward–Reverse	Amplicon length	Annealing temperature
***CCL5***	NM_001129946	CCCTGCTGTTTTTCCTACCT GCGGTTCTTTCTGGTGATAAA	117 bp	58°C
***GPX2***	DQ898282.2	GACATCAAGCGCCTCCTC AGACCAGAAAGGCAAGGTTC	182 pb	64°C
***IL22***	XM_021091968.1	TGGCAGATAACAACACAGACG GTTGGGGAACAGCACTTCTT	131 pb	58 °C
***REG3G***	NM_001144847.1	ACCCAAAACCTGGATGGATG ACCCAAAACCTGGATGGATG	102 pb	65° C
***TFF3***	NM_001243483.1	GTTGTTGCACTGCTCGGG CTCGGCTTTGTCGCTTTGT	108 pb	62°C
***HMBS***	DQ845174	AGGATGGGCAACTCTACCTG GATGGTGGCCTGCATAGTCT	83 bp	62°C
***TBP I***	DQ845178	AACAGTTCAGTAGTTATGAGCCAGA AGATGTTCTCAAACGCTTCG	153 bp	60°C

^a^*CCL5*, C-C motif chemokine ligand 5; *GPX2*, glutathione peroxidase 2; *IL22*, interleukin 22; *REG3G*, regenerating islet-derived 3γ; *TFF3*, trefoil factor 3; *HMBS*, hydroxymethylbilane synthase; *TBP*, TATA box binding protein.

### Statistics

The Affymetrix Trascripts ID’s, in general characterized each one by several exonic sequences, were associated to 13,406 Human gene names, based on Sus scrofa Ensembl (release 69, www.ensembl.org). On processed gene expression values, an exploratory functional analysis was applied with Gene Set Enrichment Analysis using the C5. BP catalog of gene sets (based on Gene Ontology) from Molecular Signatures Database v3.1 (http://www.broadinstitute.org/gsea/msigdb/Index.jsp). Normalized enrichment score (NES) was calculated for each gene set, and statistical significance was defined when False Discovery Rate % < 25 and p-values of NES < 0.05. Enrichment score p-values were estimated using a gene set-based permutation test procedure.

The overlap in enriched GO terms was visualized using the Enrichment Map (http://baderlab.org/Software/EnrichmentMap20) plugin for Cytoscape 2.8.0 (http://www.cytoscape.org/21), including the gene sets with p-value <0.005 and false discovery rate (FDR) q-value <0.10. Nodes were joined if the overlap coefficient was ≥0.5.

Finally, for all the Transcript ID’s, the fold change (as LOG2 values), the ANOVA p-value and the FDR p-value were calculated for each single comparison between the CTRL and the other loop treatments, by the pairwise procedure of Transcriptome Analysis Consolle v3.0. This procedure was used to consider the common effect of each pig on the Microarray values. Transcript ID’s were considered significant when fold change >2.00 and FDR < 0.05.

Statistical analysis of data of *REG3G* gene expression was carried out using the MIXED procedure of SAS (version 9.3; SAS Institute Inc., Cary, NC, USA). The effects of early microbiota association (against an error calculated between pigs) of loop treatment (error within pigs) and the interaction term between early microbiota association and loop treatment were tested. For the loop experiment, treatments were contrasted against control infusion. Results are presented as least-squares means and pooled SEM. Least-squares means comparisons for each interaction were made only when a tendency (p≤0.10) for an interaction between these terms was observed. Effects were considered significant at p≤0.05 and as a trend at p≤0.10.

## Results

For all the 48 samples of small intestinal tissue, the RNA purity and integrity test was passed and the microarray results were available. This allowed to perform different Gene Set Enrichment Analyses.

Considering the results of all pigs, independent of their early CA or SA association treatments after 8-h perfusion in jejunal loops with ETEC, 184 gene sets were enriched as compared with CTRL loops, whereas 74 gene sets were enriched in CTRL loops when compared to ETEC loops. On the contrary, the perfusion with LAM and F4 differentially affected a lower number of gene sets: 2 gene sets were up-regulated in LAM and F4 treatments, whereas 48 and 139 were down-regulated, respectively, as compared with CTRL. The list of the first 20 gene sets enriched with ETEC vs. CTRL, and those enriched in CTRL when compared to ETEC, LAM and F4 are reported in S1 to S4 Tables. The list of the first 50 groups of genes up-regulated or down-regulated in differently treated loops, compared to CTRL loops is reported for CA or SA treated pigs, respectively in S10 and S11 Tables, evidencing in particular the up- and down-regulated gene sets present in both CA and SA pigs inside each treatment.

**Fig 1:**
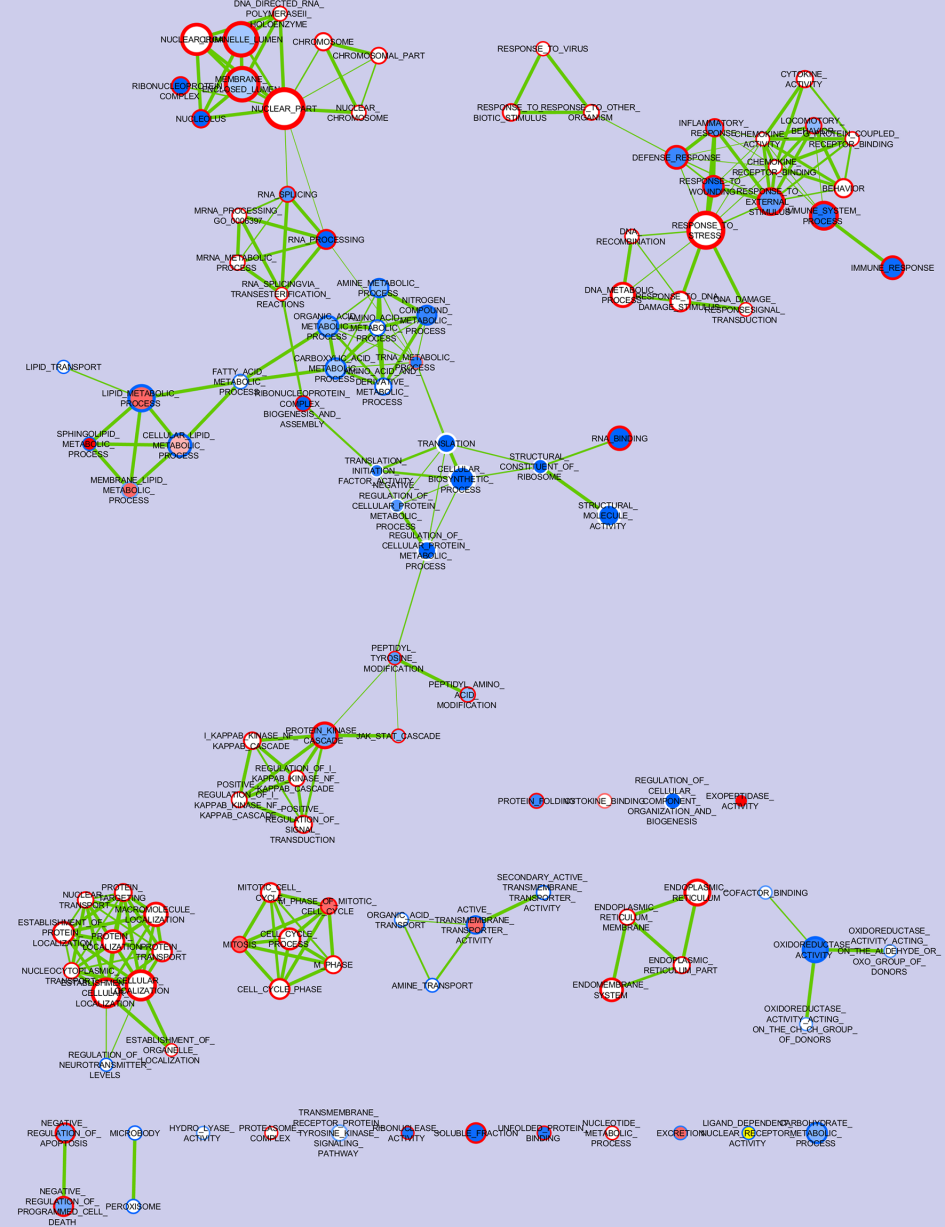
Nodes of gene sets enriched in porcine jejunal loops perfused with enterotoxigenic *Escherichia coli* (ETEC) or *Lactobacillus amylovorus* (LAM) compared to saline-perfused loops from the same subjects. Nodes represent gene-sets. Color on open circle represents the ETEC treatment, color on the center of the node circle visualizes the LAM treatment. Edges represent mutual overlap. Enrichment significance (p-value) is conveyed as node color intensity, where red is for up-regulation of ETEC or LAM treatment and blue for down-regulation. Node size represents the number of genes in the gene set.

Fig 1 visualizes the nodes of gene sets enriched (color red) or impoverished (color blue) in porcine intestinal loops perfused with ETEC (the open circle of each node) or LAM (the filled circle), compared to saline-perfused loops from the same subjects.

**Fig 2:**
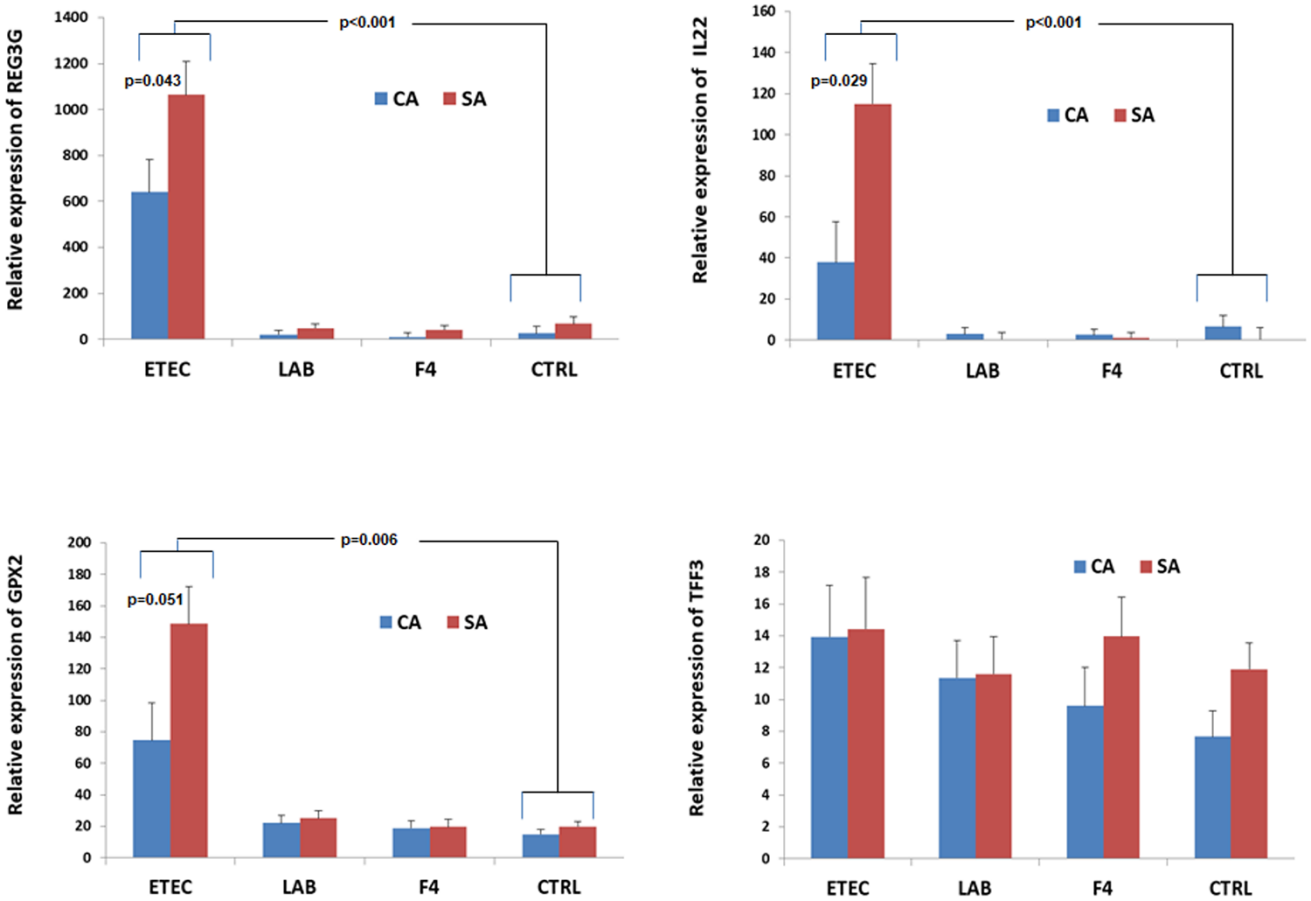
Effects of the early association with simple (SA) or complex microbiota (CA) on the relative gene expression of *REG3G*, *IL22*, *GPX2* and *TFF3* in jejunal loops perfused with ETEC, F4 fimbriae or *L*. *amylovorus*. Bars represent standard errors. A statistically significant interaction between the early treatment of pigs and the loop perfusion treatments (p = 0.018, *REG3G*; p = 0.023, *IL22*) or trend (*GPX2*, p = 0.089) was observed. The effect of loop perfusion treatments was calculated against saline treatment within each early microbiota association: ETEC, p<0.001 (*REG3G*, *IL22*) or p<0.006 (*GPX2*). Effect of early microbiota association, within the ETEC-perfused loops, p = 0.043, *REG3G*; p = 0.023, *IL22*; p = 0.051, *GPX2*; ETEC = enterotoxigenic *Escherichia coli* F4; LAM = *Lactobacillus amylovorus*; CTRL = saline.

In general there was no overlapping for gene sets enriched by ETEC and LAM. The only cases were EXOPEPTIDASE_ACTIVITY and two gene sets related to mitosis. In detail, inside the first of these gene sets, dipeptidase 1 (Renal) (*DPEP1*) was the gene ranking first among those upregulated by LAM (S6 Table), while it was downregulated by ETEC (S5 Table), compared to CTRL. The other nodes of genes enriched by LAM were related to SPHINGOLIPID_METABOLIC_PROCESS. Among the single genes that were significantly up-regulated by LAM it is important to evidence another exopeptidase gene not included in the predetermined list for EXOPEPTIDASE_ACTIVITY: carboxypeptidase O (*CPO*), and Solute Carrier Family 26 (Anion Exchanger), Member 3 (*SLC26A3*). Conversely, ETEC up-regulated several important nodes related to inflammatory response, together with cytokine activation, immune responses and DNA responses to damaging stimuli, RNA and micro RNA processing and nuclear chromosome, peptidyl-amino acid modifications and JAK-STAT cascade, cell cycling, endoplasmic reticulum and negative regulation of apoptosis. Particularly, the gene with the highest expression among those significantly upgraded by ETEC (S5 Table) was the regenerating islet-derived 3 gamma (*REG3G*) gene that encodes an antibacterial lectin with activity against Gram-positive bacteria (S5 Table). The up-regulating effect of ETEC on the *REG3G* gene was also confirmed by real-time RT PCR on the same loop samples, as compared with CTRL (Fig 2, p<0.001). Furthermore, the data showed a remarkable correlation between the loop treatments and the microbiota associations represented by the presence of a higher *REG3G* gene expression (p = 0.043) in ETEC-perfused loops of the pigs associated with the simple microbiota, compared to ETEC-perfused loops of CA pigs. The same pattern was seen also for IL22 and for GPX2. Conversely no effect of treatments was observed for the expression of *TFF3* gene, that was selected for the validation by real-time RT PCR as negative control, because although it plays a role in intestinal mucosal defense and repair, in our case was not significantly involved in gene sets more varied by the treatments.

The nodes related to metabolic processes of nitrogen compounds and oxidoreductase activity were down-regulated by both ETEC and LAM treatments. More nodes were down-regulated by LAM only, mainly related to cellular biosynthetic process, to RNA and micro RNA processing and nuclear parts; to translation and cellular biosynthetic process; to inflammatory response, cytokine activation and immune response. The particularly relevant down regulation of translational related gene sets (S3 Table) was dependent of the reduced expression of a large group of translation initiation factors (starting from early initiation factor 3 A, *EIF3A*) and of structural components of ribosomes (starting from ribosomal protein L21, *RPL21*). A few gene sets were also down-regulated in ETEC -treated loops, but they were not organized in complex nodes (with the exception of nodes related to sphingolipid metabolic process, peroxisome and to active transmembrane transporter activity of organic acids and amines). The LIGAND_DEPENDENT_NUCLEAR_RECEPTOR_ACTIVITY gene set was also down-regulated by ETEC. Beyond genes included in these sets, other genes not included and with the highest fold change were beta-carotene oxygenase 2 (*BCO2*), vanin 1 (*VNN1*) and solute carrier family 13 (sodium/sulphate symporters), member 2 (*SLC13a2*).

A second Enrichment Map was generated to evidence the nodes of gene sets that were regulated in common or separately by ETEC and F4, both compared to CTRL (S1 Fig). The results for perfusion with F4 fimbriae were similar to the results discussed for LAM, and contrasted with the effects observed after perfusion with ETEC. Thus, also with F4, several genes transcribing for early initiation factor and of structural components of ribosomes were downregulated, as compared with CTRL. In general, there was no overlapping for gene sets up-regulated by ETEC and F4 with the exception of the G_PROTEIN_COUPLED_RECEPTOR_BINDING set, were the first gene contributing to up-regulatory effect was C-C motif chemokine ligand 24 (*CCL24*). A second gene set up-regulated by F4 was the SPHINGOLIPID_METABOLIC_PROCESS, which on the contrary was down-regulated in the ETEC treatment. This was mainly related to the different activation of sphingosine-1-phosphate lyase 1 (*SGPL1*) gene.

**Fig 3:**
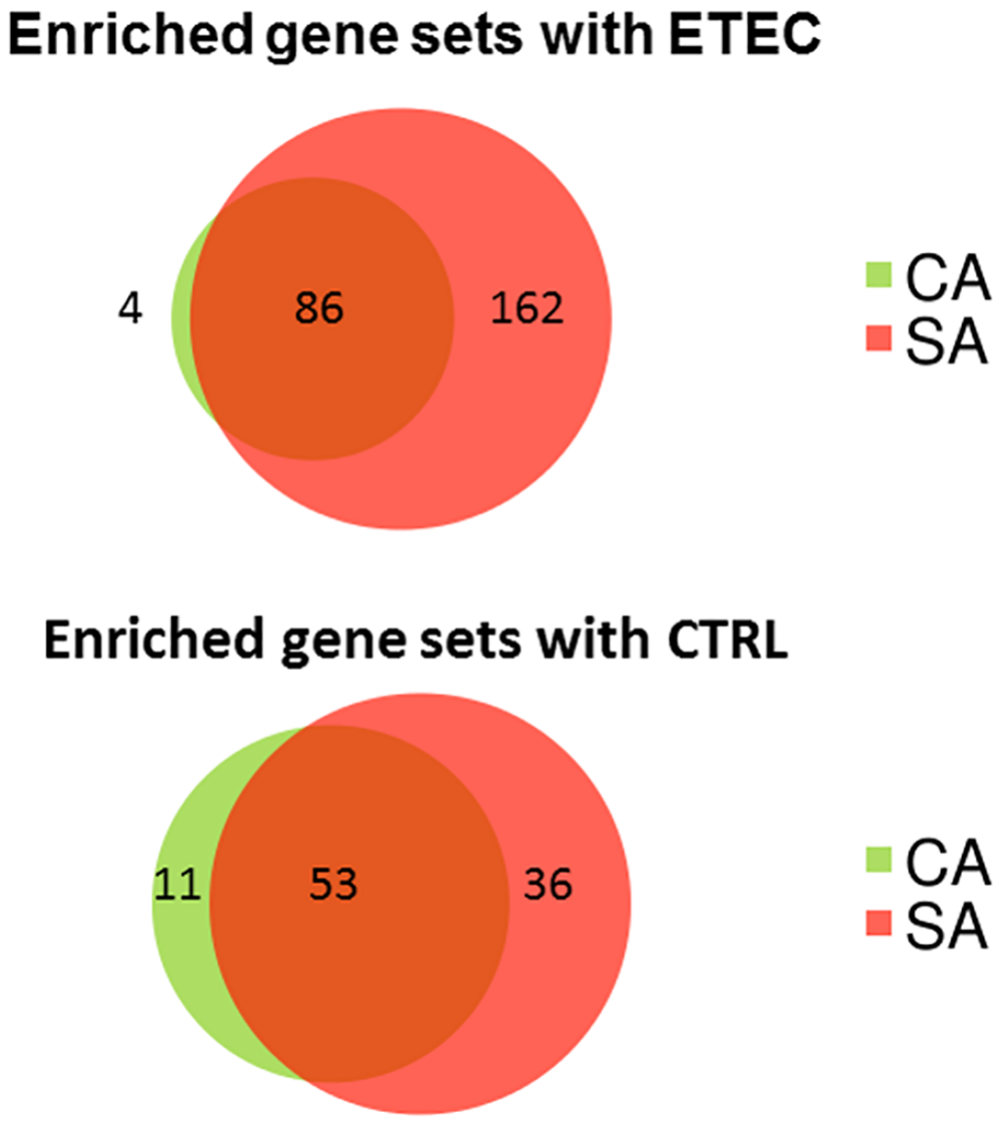
Distribution of the numbers of gene sets enriched or depleted by ETEC compared with CTRL, in the case of CA or SA pigs, or both.

**Fig 4:**
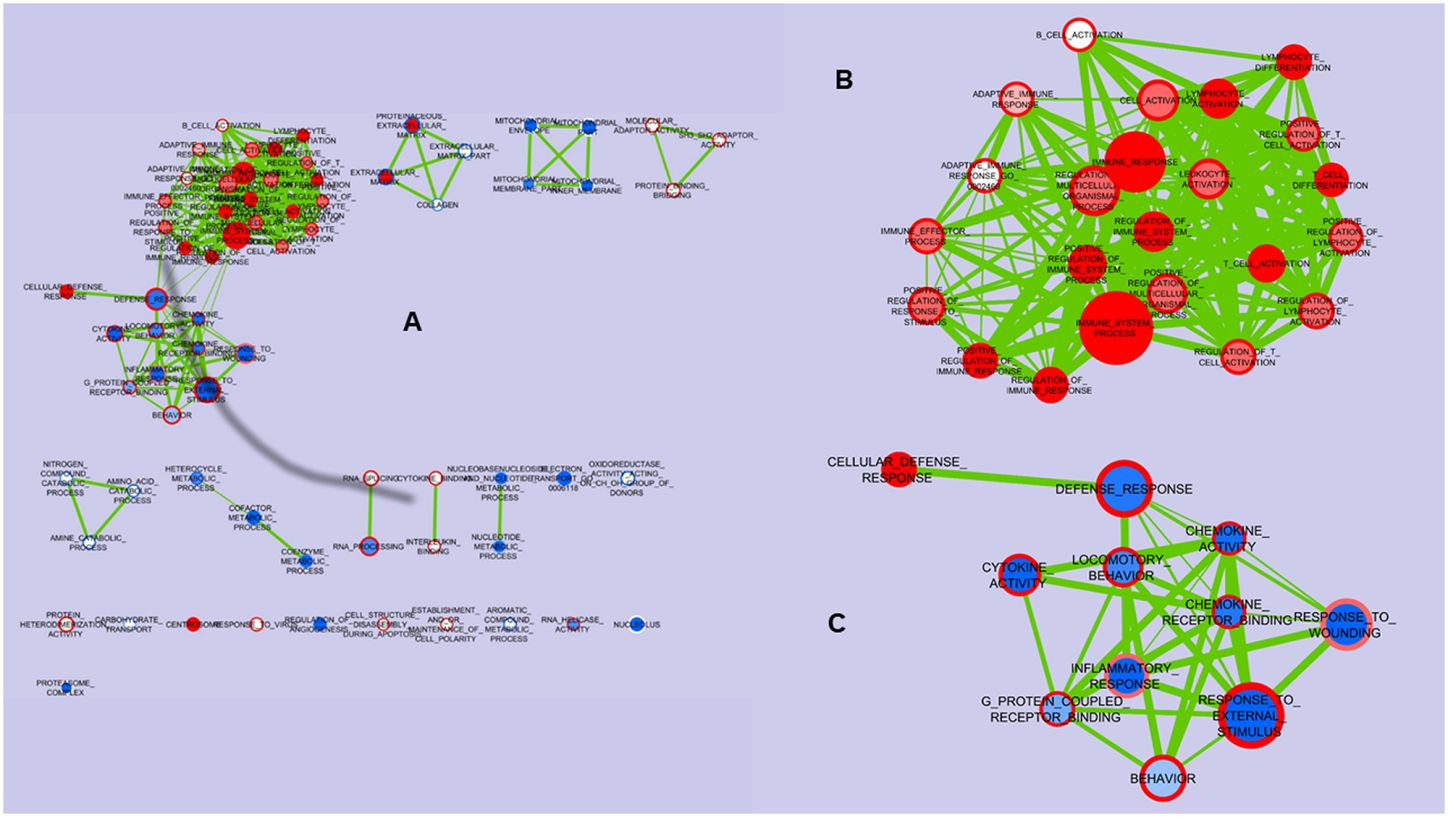
Nodes of gene sets enriched in porcine jejunal loops perfused with enterotoxigenic *Escherichia coli* (ETEC) or saline (CTRL) at 4 to 5 weeks of age, in pigs early associated with complex microbiota (CA) compared to simplex microbiota (SA) pigs. A) Whole picture; B) detail of nodes related to positive regulation of immune system processes; C) detail of nodes related to inflammatory defense response. Nodes represent gene-sets. Color on open circle of the node represents the CTRL treatment, color on the center of the node visualizes the ETEC treatment. Edges represent mutual overlap. Enrichment significance (p-value) is conveyed as node color intensity, where red is for up-regulation of ETEC or CTRL treatment in CA pigs, and blue for down-regulation, compared with SA pigs. Node size represents how many genes are in the gene-set.

With regard to the effect of early microbiota association, in CA, 57 sets were enriched compared to SA. The genes up-regulated were particularly related to the modulation and activation of lymphocytes and T cells, like *CD2*, *CD3d*, *CD4*, and *SLA2* (Src-Like-Adaptor 2) (S7 Table). Conversely, 73 gene sets were up-regulated in SA when compared to CA. They were related to various metabolic processes involving amino acids, lipids, cofactors and nucleotides, as well as to isomerase, oxidoreductase, methyltransferase or ligase activity but not to processes related to the immune system (S8 Table). The former results are averaged for the four loop treatments. Therefore, we also considered the effects of perfusion with ETEC as such. Fig 3 presents in a Venn diagram the distribution of genes sets enriched or depleted by ETEC compared with CTRL, in the case of CA or SA pigs, or both. The diagrams show that most of gene sets affected by ETEC (both upregulated and downregulated) in CA pigs were also affected in SA pigs. Conversely numerous other gene sets affected in SA pigs, were not listed for CA pigs. Fig 4A visualizes the nodes of gene sets enriched (color red) or impoverished (in blue) in intestinal loops perfused with ETEC (the filled circle of each node) or CTRL (the open circle) of CA pigs, compared to the same loop perfusions in SA pigs. This figure, in other terms, allows the simultaneous visualization of the effect of CA vs SA in ETEC treatment and in the control.

**Fig 5:**
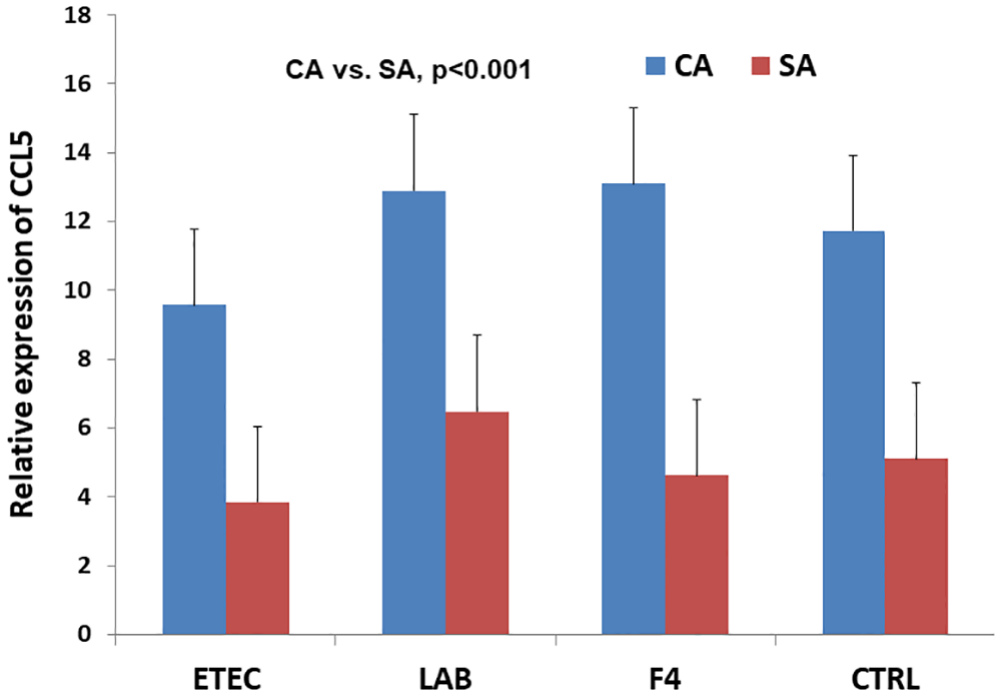
Effects of the early association with simple (SA) or complex microbiota (CA) on the relative gene expression of *CCL5* in jejunal loops perfused with ETEC, F4 fimbriae or *L*. *amylovorus*. Bars represent standard errors. Effect of early microbiota association, p<0.001; ETEC = enterotoxigenic *Escherichia coli* F4; LAM = *Lactobacillus amylovorus*; CTRL = saline.

The early association of piglets with a complex microbiota, compared to a simpler microbiota, resulted in an up-regulation of a large number of gene sets that were related to the regulation and activation of lymphocytes, above all of T cells, as well as to cellular defense responses both in ETEC and in CTRL loops (Fig 4B and 4C). Genes for chemokine and cytokine activity, and in general for response to external stimulus, were down-regulated in ETEC loops obtained from CA pigs, compared to those coming from SA pigs (Fig 4C). Conversely, the same gene sets were up-regulated in CTRL loops from CA pigs, compared to those from SA pigs. Nevertheless, in both cases, the gene sets involved were not among the first ranking gene sets in the list, and the single genes that explained the interactive effect for chemokine and cytokine activity related sets were not the same. For example the most representative gene for the down-regulation in ETEC loops obtained from CA pigs, vs SA pigs, was interleukin 1 receptor antagonist (*IL1RN*), while the most representative gene for the up-regulation in CTRL loops obtained from CA pigs, vs SA pigs, was *CCL5*. This was also confirmed by RT-qPCR for this gene (Fig 5).

The interaction of LAM and CTRL with CA or SA treatment was much less evident (S2 Fig). It can be only observed that the association of the intestinal loop with LAM reduced the number of gene sets that were differentially regulated by the early association of pigs with complex or simple microbiota, compared with CTRL. The picture was very similar also for F4 (data not shown).

## Discussion

Several factors drive the establishment of the microbial community in the porcine gut [3, 18], particularly during the first days of life. In an earlier experiment, CD piglets that were orally dosed with an inoculant consisting of sow’s diluted feces in the first days after birth had a more complex intestinal and fecal microbiota for the first four weeks of life, compared to those receiving only the same SA treatment as in the present study [19]. The process of formation of the consortium of gut microbiota is considered to be age-dependent, stimulated by new environmental stimuli, and mutually controlled by the intervention of the mucosa-associated lymphoid tissue (MALT) [20]. A delayed or too precocious encounter with environmental microbes may switch the overall gut microbiota profile and affect the gut maturation. Particularly, the activation of dendritic cells by microbiota-derived ligands promotes spontaneous T cell proliferation [21]. In conventionally-reared piglets of 3 to 4 weeks of age, CD4+ T cells progressively colonize the intestinal mucosa, the number of intraepithelial lymphocytes raises, while IgM secreting cells are appearing [22]. A complex activation of a large array of cytokines and chemokines marks this process. The early association with a microbiota of porcine origin was signed at this age (3 weeks) by the general average upregulation of the expression of gene sets of cytokines and chemokines and of their receptors in the porcine stomach, compared with pigs where the early association was limited to a bacteria mixture restricted to three commensal bacteria [23]. Here we show that oral administration of a culture of a few microbial species in the early phase of life in a high hygiene environmental condition may not be sufficient for the newborn piglet to activate T lymphocytes in the small intestine few weeks later. Conversely, T cell activation was more stimulated after association with a complex microbiota, represented by a fecal bacterial community obtained from a mature sow. Thus it was favored a more timely maturation of the MALT. In the stressful immediate post-weaning phase, an inefficient or insufficient activation of T cells may contribute to the local damage typically observed in intestinal mucosa of piglets [24]. In association with an improved T cell activation/regulation, a more complex microbiota stimulated also the transcription of several receptor and adaptor molecules (firstly chemokine -C-C Motif- ligand 5 –Fig 5 -, and T cell receptor associated transmembrane adaptor 1) related to cellular defense response. This group represents a set of markers for innate lymphoid cells involved in the regulation of host-commensal bacteria interactions during inflammation [25]. It is relevant that the *ITGAE* gene (Integrin, alpha E—antigen CD103—human mucosal lymphocyte antigen 1; alpha polypeptide), which is preferentially expressed in intestinal intraepithelial lymphocytes [26] and intestinal dendritic cells [27], was ranked seventh in the list of the most up-regulated genes (S9 Table). Furthermore, the induction and function of this integrin is linked to the activation of another gene that is in the same list C-C motif chemokine receptor 9 (*CCR9*) [28], in responding T cells, in the lamina propria and mesenteric lymph nodes. Taken together, these data evidence a role of early microbial association in the local recruitment of immune cells in MALT.

With the perfusion with ETEC, compared to control treatment, many more gene sets were upregulated in piglets early associated with a simplified microbiota compared to those associated to a complex microbiota (Fig 3). Particularly the first showed an increased activation of genes related to inflammation, such as chemokine and cytokine activity, and of genes related to responses to external stimuli in general, in loops perfused with ETEC, compared to CA piglets. This again may be explained by the different timing of the maturation of the MALT. An adequate stimulation of toll-like receptors (TLRs) by commensal microbiota promotes intestinal homeostasis [28] and this presumably favored the limitation of the diffusion of inflammation with the starting of the ETEC infection in CA pigs. The reverse was in control loops that is that CA pigs had relatively more upregulated gene sets related to chemokine and cytokine activity, than SA pigs. This is apparently quite surprising, but it could be related to the higher bacteria diversity in CA pigs, as observed in the preceding research on CD pigs with different microbe association [19]. In fact, more diverse commensal microbes may require more activation of genes related to response to external stimuli, as indicated by the up regulation of this gene set in control loops of CA pigs. Thus it can be concluded that pigs early associated to a complex microbiota had control jejunal loops in a more activated condition, able to respond rapidly to possible infections.

Nevertheless, TLR signaling is important to induce also inflammatory responses by several cytokines [29]. Thus, other interacting factors may explain the different response to ETEC depending on the type of the early microbiota association. In ETEC-perfused loops of SA pigs, as compared with CA, the most up-regulated gene within the set responsible for inflammatory response was interleukin 1 alpha (*IL1A*). In recent studies targeting Crohn’s disease in humans, it was evidenced that the kinase MAP3K4 (Mitogen-activated protein kinase 4) was responsible for variation of IL1A, regardless of variation in TLR activation [30]. The *MAP3K4* gene was also up-regulated in ETEC-perfused loops of SA pigs, as compared with CA. Furthermore, a different modulation of immune response related to IL1A was also expected depending on the down-regulation of *IL1RN* in ETEC loops obtained from CA pigs, vs SA pigs, due to the inhibitory activity of interleukin-1 action by the binding of the product of this gene to the IL1A receptor. Unfortunately, no other study has addressed this topic and more research is required to verify the relevance of this observation for gut health later in the life of differently microbe-associated pigs. Villus height and crypt depth are considered markers of the health status of the mucosa. In our previous report, based on the same study [31], no effect of the early association type was seen on villus height and crypt depth nor a correlation of association treatment with loop perfusion treatments. Conversely, we showed a significant decrease of villous height and villous surface in ETEC challenged loops compared to CTRL, indicating a worsening of functional status of the jejunal mucosa. This is in agreement with the findings of the present study related to the comparison of ETEC perfused loops to CTRL (S2 Table), that evidenced that the first gene set down-regulated by ETEC was involved in transmembrane transport activity pathways, as did several other related genes sets. A lower expression of the genes related to absorption capacity is clearly related to a lower intestinal epithelial functionality and could be associated to a low intestinal health status induced by ETEC challenge. However, in a parallel study that included also the pigs used in the present experiment, CA treatment did not prevent the reduction of gut net fluid absorption or gut permeability for fluorescein sodium salt in intestinal loops perfused with ETEC compared to CTRL loops [32]. Overall, this may indicate that the time of perfusion with ETEC was sufficient to evidence a mild effect of the different early microbial association of pigs at transcriptional and morphological level, but this was not sufficient to differentially affect the permeability of the mucosa.

Here we applied the SISP approach to CD piglets about four weeks after early oral association to a simple or complex microbiota in order to compare the effects of an enterotoxigenic pathogen (ETEC) and a commensal (LAM) strain on the jejunal transcriptome. Furthermore, our approach allowed comparing the perfusion treatment effects in the same piglets, limiting the effects of genetic variability. A conspicuous number of genes and gene sets was affected after 8 hours of jejunal perfusion with ETEC compared with saline perfusion. This was not unexpected based also on previous literature on the topic [6, 33–34]. Since our results were based on the use of a second generation microarray system that rests upon the integration of several exon probes for each gene, an even more detailed and reliable description of the transcriptome changes associated with the ETEC challenge was presumably obtained.

Among the genes already evidenced as markers of ETEC infection, *REG3G* is probably the most signaled in literature. The specific antimicrobial protein encoded by *REG3G* is released by different types of small intestinal cells during infection but under prolonged stimulation its presence is depleted due to prolonged release [35]. Thus, an up-regulated production of the *REG3G* transcripts seems reasonable in the early stage of infection. Furthermore, we also found that the myeloid differentiation primary response 88 gene (*MYD88*) was up-regulated by ETEC (FDR = 0.003); the MYD88 protein, among its several actions related to pathways connecting the recognition of bacteria and inflammatory response, also regulates the expression of *REG3G* [36]. Notably pigs that were early associated to a simple microbiota had a higher expression of *REG3G* in ETEC-perfused loops. However, *MYD88* was equally up-regulated by ETEC in both groups of pigs differently associated at early age. Another related gene that was sharply up-regulated by ETEC was *IL22* (Fig 2 and S1 Table). This interleukin was recently indicated as being secreted by group 3 innate lymphoid cells located in the intestinal crypts, during bacterial damage or T lymphocyte trigger [37]. Furthermore, small intestinal organoids cultured with IL22 increased *REG3G* mRNA production and tissue regeneration [38].

Research has evidenced that the beneficial microbe *Akkermansia muciniphila* is able to restore the production of REG3G [39], but only after gut disturbance induced by a high fat diet. In the present study, LAM did not affect the *REG3G* gene expression, which can be explained by the fact that healthy pigs were used and fed with a low fat diet.

The several-fold reduced expression of *SLC13A1* and *VNN1* by ETEC may have implications for the intestinal barrier defense. The *SLC13A1* encoded protein, necessary for sulphate (re)absorption, is important for mucin sulfonation [40]. *VNN1* is a redox-sensitive gene coding for a pantetheinase that releases the antioxidant cysteamine [41]. VNN1 has also a complex and multi-faced role for the control of intestinal inflammation that, however, still needs to be more fully clarified [42]. In fact, pantetheine has antibacterial activity against *E*. *coli*, and it can be presumed that this molecule can be better protected from hydrolysis [43] when *VNN1* is down-regulated by ETEC. The impact of ETEC on the redox status was also confirmed by the sharp upregulation observed in *GPX2* gene, presumably stimulated by hydrogen peroxide resulting from inflammation. This may imply an increased production of the selenoprotein product and thus a specific increased dietary selenium requirement.

Another observation, important for possible implications related to the nutrient requirements of growing pigs and in general for pig production in practice, is that the gene set LIGAND_DEPENDENT_NUCLEAR_RECEPTOR_ACTIVITY was up-regulated in CTRL, compared to ETEC. In particular, the genes down-regulated by ETEC were constitutive androstane receptor–formally the nuclear receptor subfamily 1, group I, member 3—(*NR1I3*), thyroid hormone receptor beta (*THRb*), retinoid X receptor alpha (*RXRa*), vitamin D receptor (*VDR*), and mineral corticoid receptor–formally nuclear receptor subfamily 3, group C, member 2 (*NR3c2*). LPS is an inhibitor of androstane receptor, based on the comparison of gene expression in LPS-treated wild-type mice and LPS-treated NR1I3-null mice [44], and was also seen to reduce hepatic *RXRa* gene expression [45]. Thus, the increased presence of LPS from ETEC in the loops can explain the differential regulation of the gene set LIGAND_DEPENDENT_NUCLEAR_RECEPTOR_ACTIVITY. Remarkably, the reduction of the gene expression of the nuclear receptors of vitamin A and D during ETEC perfusion may imply an increased dietary requirement for these vitamins to preserve the regulatory efficacy on RNA transcription, when pigs are stressed by ETEC infection.

The effects of the perfusion of LAM on the transcriptome of jejunal loops were generally mild. However, the presence of the *SLC26A3* gene among the genes most up-regulated by the perfusion with this microbe is important. *SLC26A3*, also known as *DRA* (Congenital Chloride Diarrhea), encodes a protein typically expressed in enterocytes that contributes to the chloride reabsorption. The raise of intracellular cyclic AMP or GMP, induced by several bacterial enterotoxins, is responsible for chloride loss and watery diarrhea via the activation of apical chloride channels [46]. Thus, the triggering of the *SLC26A3* gene by LAM may compensate at least in part the endotoxin-induced activation of chloride channels and may reduce the occurrence of diarrhea. This can explain the small improvement of villus height in LAM-perfused loops [31]. Also *Lactobacillus acidophilus* stimulated the expression of *SLC26A3* via a transcriptional mechanism [47]. Interestingly, among the first genes downregulated by LAM was cystic fibrosis transmembrane conductance regulator (CFTR) (seventh in the ranking order produced by Gene Set Enrichment Analysis). CFTR is a cAMP- and phosphorylation-regulated chloride/bicarbonate channel, regulating the chloride efflux; thus, its activation is involved in enterotoxin-induced secretory diarrhea [48]. Both these gene regulations could have contributed to the favorable response observed in the oral supplementation with LAM in ETEC-challenged weaning pigs [9].

The reduced expression of a large group of translation initiation factors and of structural components of ribosomes observed in LAM loops, may also reflect a reduced pressure of the intestinal epithelium to produce new proteins. This could apparently conflict with the observed promotion of intestinal epithelial cell turnover by commensal bacteria [49]. However, LAM is a normal guest of the pig gut, it was also provided to SA piglets with early microbiota association, and thus it could have created a sort of tolerogenic condition in the LAM-perfused intestinal loop.

Finally, the villus height of LAM loops could have been improved by the better ability to hydrolyze dipteptides at the enterocyte surface, thanks to the up-regulation of *DPEP2*, transcribing for a glycolipid-anchored ectoenzyme [50], that furthermore was conversely down-regulated in ETEC loops.

The purpose of including a separate loop perfusion with F4, in addition to ETEC perfusion, was to have the opportunity to separate the effect of F4 on jejunum mucosa from the pathogenic effect of ETEC toxins. The pattern of changes in transcriptome expression after perfusion with purified F4 fimbriae did not resemble that observed after ETEC perfusion. Oral provision of F4 fimbriae in pigs was able to stimulate immune responses in ETEC-susceptible pigs [51]. In fact, endocytosis and translocation of F4 fimbriae across the gut epithelium, with the appearance of fimbriae in the lamina propria and dome region induces protective mucosal immunity upon ETEC infection [52]. In our study, however, the dose was lower and the limited exposure time was presumably insufficient to activate a response, as compared to tests performed to assess the utility of F4 for oral vaccination of pigs [51]. Conversely, a large group of translation initiation factors and of structural components of ribosomes was found to be rich of genes down-regulated by F4, as it was observed for LAM. No other research related to the F4 specific effect on transcriptome was found in literature, except studies related to its possible use as a vaccine [51], and the down regulation of genes related to the translation machinery is difficult to be explained. Nevertheless, the present results may indicate that before the eventual raise of ETEC- specific immune response, no short-term unfavorable response at transcriptional level should be recorded upon the oral provision of F4. This may also be useful considering that F4 or similar protein structures could also be considered as a tool to protect against ETEC adhesion competitively, as already recently shown in vitro, in comparison with some galactose-binding lectin of vegetable origin [53].

## Conclusions

The present experiment evidences that two functionally distinct small intestinal states are detectable with different early encounter of microbes by the young pig. An early association with a complex microbiota is able to up-regulate gene sets related to the modulation and activation of B and T lymphocytes, when compared to an association with a simple microbiota, the effect being maintained during intestinal ETEC challenge. Furthermore, piglets early associated with a complex microbiota maintain a reduced activation of genes related to chemokine and cytokine activity after ETEC challenge. This may indicate that early microbial colonization is important for the establishment of balanced immune and inflammatory responses under both enterotoxigenic pathogen challenge and non-challenged conditions, but needs to be confirmed with further studies oriented to assess the implications for the permeability of the mucosa. Overall this implies that more attention should be given to the farming practices allowing the suckling newborn pig to experience rapid contacts with a complex microbiota and to a rapid immune programming.

Intestinal ETEC challenge powerfully affects the expression of genes already in the early infection phase, inducing strong and typical immune and inflammatory responses. Conversely the perfusion with purified ETEC F4 fimbriae is not sufficient to activate the whole inflammatory cascade induced by live ETEC bacteria, and the transcriptome results indicate that it could be well tolerated in case of oral supplementation.

The additional presence of *Lactobacillus amylovorus* in the jejunal lumen is rapidly characterized by the down-regulation of several pathways related to RNA and translation. Together with the down-regulation of gene sets related to inflammatory and immune responses and to cellular compound metabolic processing, induced by *Lactobacillus amylovorus*, this confirms the interest for this pig commensal microbe to be considered as a potential probiotic for pigs, particularly to reduce the metabolic costs of the co-habitation with gut microbiota and improve feed efficiency.

## Supporting information

S1 Fig Nodes of gene sets enriched in porcine jejunal loops perfused with enterotoxigenic *Escherichia coli* (ETEC) or ETEC F4 fimbriae (F4), at 4 to 5 weeks of age, compared to saline-perfused loops from the same subjects. Nodes represent gene-sets. Color on ring of the node represents the ETEC treatment, color on center visualizes the F4 treatment. Edges represent mutual overlap. Enrichment significance (p-value) is conveyed as node color intensity, where red is for up-regulation of ETEC or F4 treatment and blue for down-regulation. Node size represents how many genes are in the gene-set.
(TIF)

S2 Fig Nodes of gene sets enriched in porcine jejunal loops perfused with *Lactobacillus amylovorus* (LAM) or saline (CTRL) at 4 to 5 weeks of age, in pigs early associated with complex microbiota (CA) compared to simplex microbiota (SA) pigs. Nodes represent gene-sets. Color on ring of the node represents the CTRL treatment, color on center visualizes the LAM treatment. Edges represent mutual overlap. Enrichment significance (p-value) is conveyed as node color intensity, where red is for up-regulation of CTRL or LAM treatment in CA pigs, and blue for down-regulation, compared with SA pigs. Node size represents the number of genes in the gene set.
(TIF)

S1 Table Ordered list of the first twenty groups of genes up-regulated in ETEC treated loops, compared to CTRL loops. NES = normalized enrichment score; FDR = false discovery rate.
(DOCX)

S2 Table Ordered list of the first twenty groups of genes down-regulated in ETEC treated loops, compared to CTRL loops. NES = normalized enrichment score; FDR = false discovery rate.
(DOCX)

S3 Table Ordered list of the first twenty groups of genes up-regulated in LAM treated loops, compared to CTRL loops. NES = normalized enrichment score; FDR = false discovery rate.
(DOCX)

S4 Table Ordered list of the first twenty groups of genes up-regulated in F4 treated loops, compared to CTRL loops. NES = normalized enrichment score; FDR = false discovery rate.
(DOCX)

S5 Table Statistically significant genes (false discovery rate, P<0.05) for the pairwise contrast ETEC vs. CTRL, ordered for fold change. n.a.: not assigned.
(DOCX)

S6 Table Statistically significant genes (false discovery rate, P<0.05) for the pairwise contrast LAM vs. CTRL, ordered for fold change. n.a.: not assigned.
(DOCX)

S7 Table Ordered list of the first twenty groups of genes up-regulated in CA treated pigs, compared to SA pigs. NES, normalized enrichment score; FDR, false discovery rate.
(DOCX)

S8 Table Ordered list of the first twenty groups of genes up-regulated in CA treated pigs, compared to SA pigs. NES, normalized enrichment score; FDR, false discovery rate.
(DOCX)

S9 Table Ordered list of the first ten statistically significant genes (false discovery rate–FDR-, P<0.05) up-regulated in CA treated pigs, compared to SA pigs. (DOCX)

S10 Table List of the first fifty groups of genes up-regulated in differently treated loops, compared to CTRL loops and in CA or SA treated pigs. Up-regulated gene sets present in both CA and SA pigs inside each treatment are colored in yellow.
(DOCX)

S11 Table List of the first fifty groups of genes down-regulated in differently treated loops, compared to CTRL loops and in CA or SA treated pigs. Down-regulated gene sets present in both CA and SA pigs inside each treatment are colored in yellow.
(DOCX)
