# Supplementary figures and images for: Molecular networks affected by neonatal microbial colonization in porcine jejunum, luminally perfused with enterotoxigenic Escherichia coli, F4ac fimbria or Lactobacillus amylovorus
Source: PLoS One. 2018 Aug 30;13(8):e0202160. doi: 10.1371/journal.pone.0202160 (PMC6116929; doi:10.1371/journal.pone.0202160)

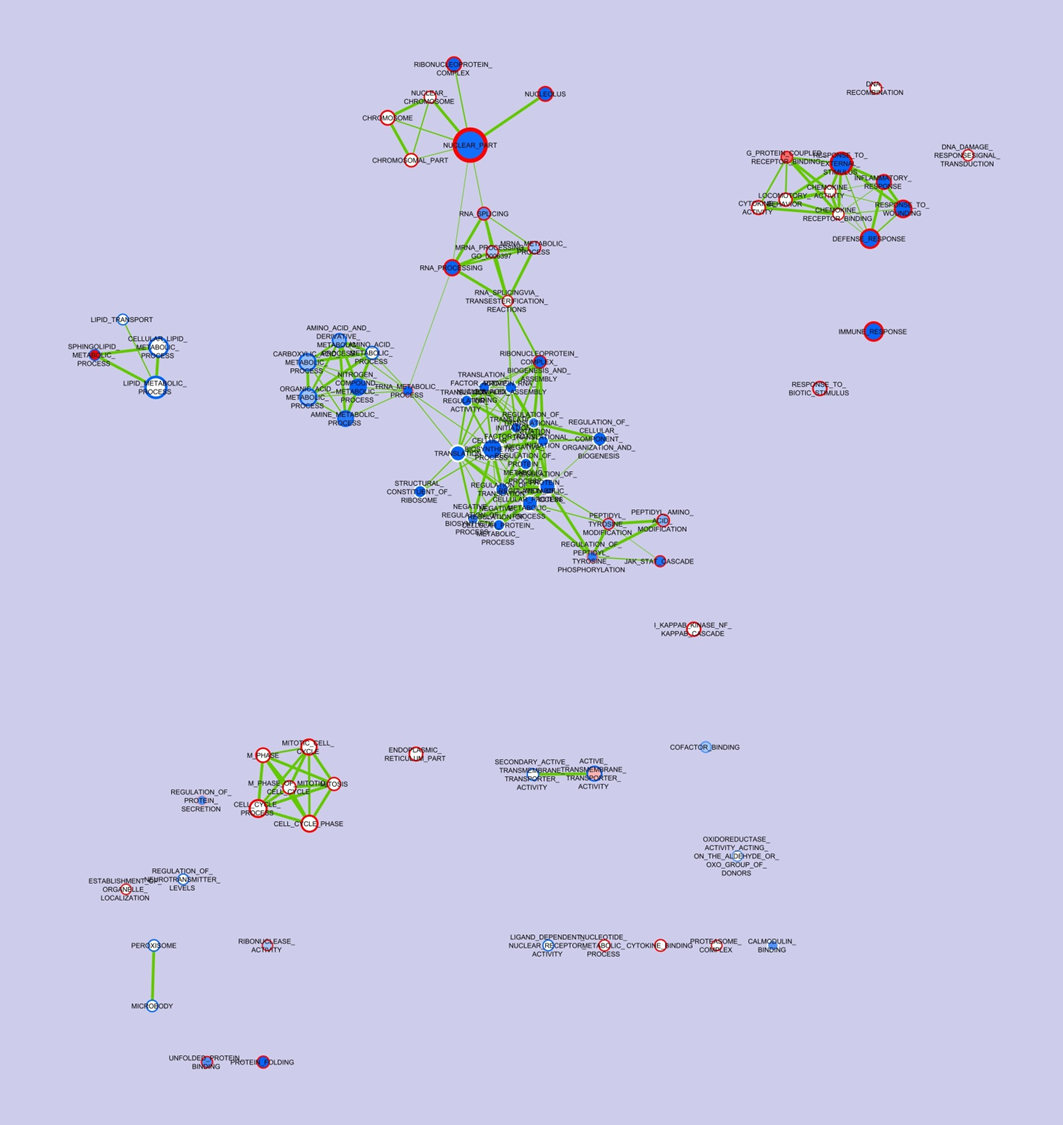

Supplement: S1 Fig — Nodes represent gene-sets. Color on ring of the node represents the ETEC treatment, color on center visualizes the F4 treatment. Edges represent mutual overlap. Enrichment significance (p-value) is conveyed as node color intensity, where red is for up-regulation of ETEC or F4 treatment and blue for down-regulation. Node size represents how many genes are in the gene-set. (TIF) [file pone.0202160.s001.tif]

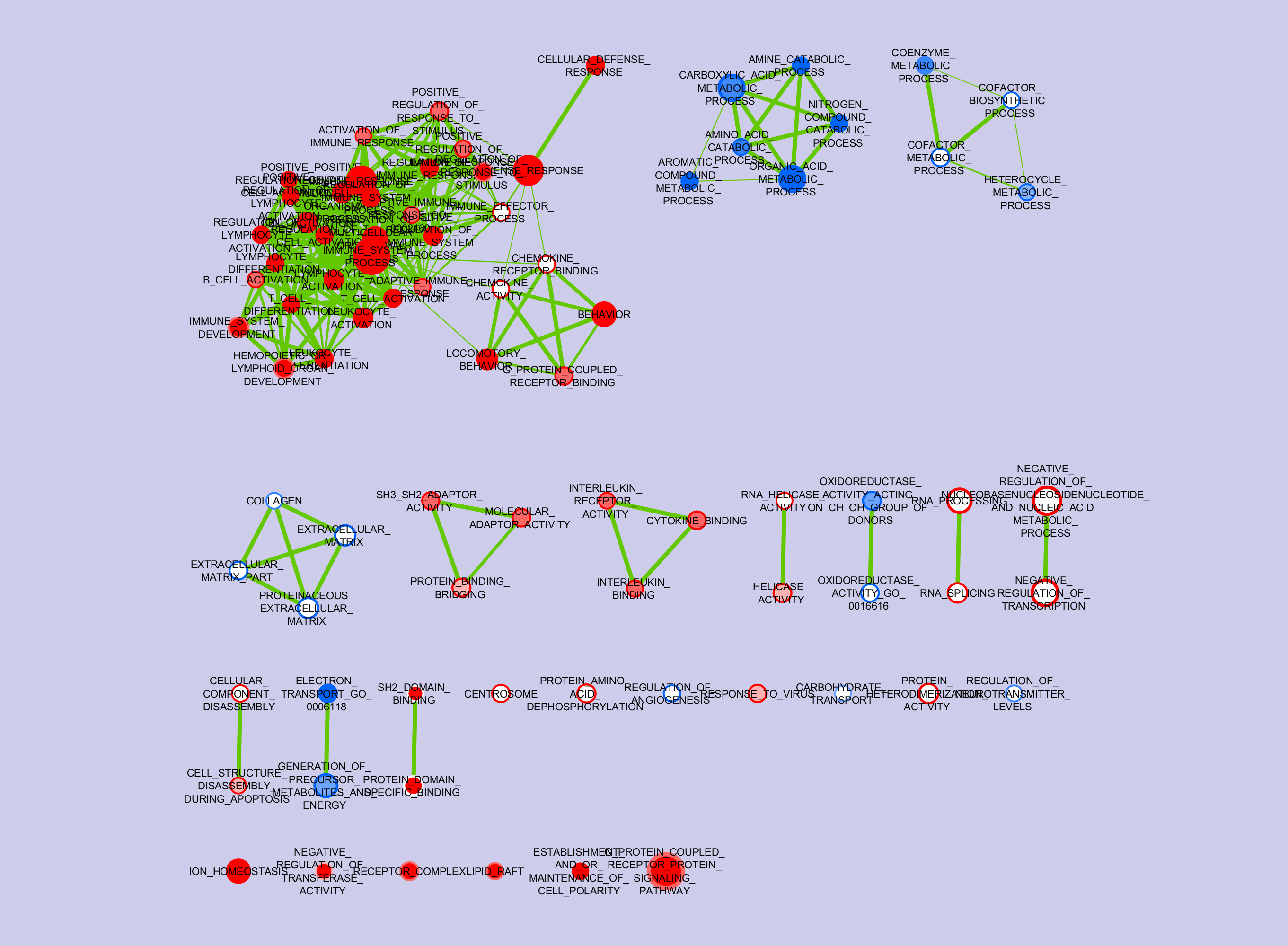

Supplement: S2 Fig — Nodes represent gene-sets. Color on ring of the node represents the CTRL treatment, color on center visualizes the LAM treatment. Edges represent mutual overlap. Enrichment significance (p-value) is conveyed as node color intensity, where red is for up-regulation of CTRL or LAM treatment in CA pigs, and blue for down-regulation, compared with SA pigs. Node size represents the number of genes in the gene set. (TIF) [file pone.0202160.s002.tif]
