# Supplementary material for: Molecular networks affected by neonatal microbial colonization in porcine jejunum, luminally perfused with enterotoxigenic Escherichia coli, F4ac fimbria or Lactobacillus amylovorus
Source: PLoS One. 2018 Aug 30;13(8):e0202160. doi: 10.1371/journal.pone.0202160 (PMC6116929; doi:10.1371/journal.pone.0202160)
Supplement: S1 Table — NES = normalized enrichment score; FDR = false discovery rate. (DOCX) [file pone.0202160.s003.docx]

**S1 Table.** **Ordered list of the first twenty groups of genes up-regulated in ETEC treated loops, compared to CTRL loops (NES, normalized enrichment score; FDR, false discovery rate).**

| NAME | SIZE | NES | FDR q-val |
| --- | --- | --- | --- |
| RNA_PROCESSING | 122 | 2.336 | 0.000 |
| RNA_SPLICING | 63 | 2.245 | 0.000 |
| PROTEASOME_COMPLEX | 18 | 2.197 | 0.000 |
| RIBONUCLEOPROTEIN_COMPLEX | 101 | 2.031 | 0.010 |
| CHEMOKINE_ACTIVITY | 28 | 2.018 | 0.009 |
| MRNA_PROCESSING_GO_0006397 | 45 | 1.993 | 0.012 |
| CHEMOKINE_RECEPTOR_BINDING | 29 | 1.967 | 0.015 |
| JAK_STAT_CASCADE | 29 | 1.964 | 0.013 |
| CYTOKINE_ACTIVITY | 82 | 1.946 | 0.015 |
| LOCOMOTORY_BEHAVIOR | 68 | 1.925 | 0.018 |
| RESPONSE_TO_BIOTIC_STIMULUS | 86 | 1.925 | 0.016 |
| PEPTIDYL_AMINO_ACID_MODIFICATION | 54 | 1.916 | 0.016 |
| DEFENSE_RESPONSE | 180 | 1.901 | 0.020 |
| MRNA_METABOLIC_PROCESS | 53 | 1.897 | 0.019 |
| ESTABLISHMENT_OF_ORGANELLE_LOCALIZATION | 15 | 1.897 | 0.018 |
| G_PROTEIN_COUPLED_RECEPTOR_BINDING | 37 | 1.891 | 0.018 |
| INFLAMMATORY_RESPONSE | 97 | 1.880 | 0.019 |
| RESPONSE_TO_WOUNDING | 146 | 1.874 | 0.019 |
| PROTEIN_FOLDING | 48 | 1.854 | 0.024 |
| NUCLEAR_CHROMOSOME | 43 | 1.849 | 0.024 |
