# Supplementary material for: Molecular networks affected by neonatal microbial colonization in porcine jejunum, luminally perfused with enterotoxigenic Escherichia coli, F4ac fimbria or Lactobacillus amylovorus
Source: PLoS One. 2018 Aug 30;13(8):e0202160. doi: 10.1371/journal.pone.0202160 (PMC6116929; doi:10.1371/journal.pone.0202160)
Supplement: S2 Table — NES = normalized enrichment score; FDR = false discovery rate. (DOCX) [file pone.0202160.s004.docx]

**S2 Table. Ordered list of the first twenty groups of genes down-regulated in ETEC treated loops, compared to CTRL loops (NES, normalized enrichment score; FDR, false discovery rate).**

| NAME | SIZE | NES | FDR q-val |
| --- | --- | --- | --- |
| SECONDARY_ACTIVE_TRANSMEMBRANE_TRANSPORTER_ACTIVITY | 37 | -2.111 | 0.003 |
| SPHINGOLIPID_METABOLIC_PROCESS | 23 | -1.974 | 0.020 |
| LIGAND_DEPENDENT_NUCLEAR_RECEPTOR_ACTIVITY | 22 | -1.957 | 0.019 |
| ORGANIC_ACID_METABOLIC_PROCESS | 144 | -1.945 | 0.017 |
| CARBOXYLIC_ACID_METABOLIC_PROCESS | 143 | -1.917 | 0.021 |
| AMINO_ACID_AND_DERIVATIVE_METABOLIC_PROCESS | 80 | -1.899 | 0.023 |
| SYMPORTER_ACTIVITY | 25 | -1.889 | 0.023 |
| LIPID_TRANSPORT | 22 | -1.857 | 0.031 |
| PEROXISOME | 37 | -1.836 | 0.036 |
| MICROBODY | 37 | -1.833 | 0.034 |
| NITROGEN_COMPOUND_METABOLIC_PROCESS | 123 | -1.802 | 0.047 |
| LIPID_METABOLIC_PROCESS | 234 | -1.802 | 0.043 |
| OXIDOREDUCTASE_ACTIVITY_ACTING_ON_THE_ALDEHYDE_OR_OXO_GROUP_OF_DONORS | 16 | -1.785 | 0.049 |
| ACTIVE_TRANSMEMBRANE_TRANSPORTER_ACTIVITY | 98 | -1.782 | 0.047 |
| CELLULAR_LIPID_METABOLIC_PROCESS | 184 | -1.780 | 0.045 |
| COFACTOR_BINDING | 22 | -1.776 | 0.044 |
| AMINE_METABOLIC_PROCESS | 113 | -1.776 | 0.042 |
| REGULATION_OF_NEUROTRANSMITTER_LEVELS | 21 | -1.757 | 0.049 |
| COENZYME_BINDING | 16 | -1.754 | 0.048 |
| AMINO_ACID_METABOLIC_PROCESS | 65 | -1.752 | 0.046 |
