# Supplementary material for: Molecular networks affected by neonatal microbial colonization in porcine jejunum, luminally perfused with enterotoxigenic Escherichia coli, F4ac fimbria or Lactobacillus amylovorus
Source: PLoS One. 2018 Aug 30;13(8):e0202160. doi: 10.1371/journal.pone.0202160 (PMC6116929; doi:10.1371/journal.pone.0202160)
Supplement: S3 Table — NES = normalized enrichment score; FDR = false discovery rate. (DOCX) [file pone.0202160.s005.docx]

**S3 Table.** **Ordered list of the first twenty groups of genes down-regulated in LAB treated loops, compared to CTRL loops (NES, normalized enrichment score; FDR, false discovery rate).**

| NAME | SIZE | NES | FDR q-val |
| --- | --- | --- | --- |
| STRUCTURAL_CONSTITUENT_OF_RIBOSOME | 56 | -2.471 | 0.000 |
| RIBONUCLEOPROTEIN_COMPLEX_BIOGENESIS_AND_ASSEMBLY | 58 | -2.258 | 0.000 |
| TRANSLATION | 131 | -2.172 | 0.001 |
| RIBOSOME_BIOGENESIS_AND_ASSEMBLY | 14 | -2.148 | 0.001 |
| TRNA_METABOLIC_PROCESS | 16 | -2.050 | 0.007 |
| RRNA_METABOLIC_PROCESS | 12 | -2.030 | 0.008 |
| CELLULAR_BIOSYNTHETIC_PROCESS | 241 | -1.990 | 0.013 |
| TRANSLATION_INITIATION_FACTOR_ACTIVITY | 17 | -1.890 | 0.053 |
| REGULATION_OF_CELLULAR_COMPONENT_ORGANIZATION_AND_BIOGENESIS | 93 | -1.880 | 0.053 |
| RNA_BINDING | 190 | -1.857 | 0.063 |
| NUCLEOLAR_PART | 14 | -1.854 | 0.059 |
| REGULATION_OF_CELLULAR_PROTEIN_METABOLIC_PROCESS | 121 | -1.839 | 0.064 |
| NEGATIVE_REGULATION_OF_CELLULAR_PROTEIN_METABOLIC_PROCESS | 36 | -1.809 | 0.087 |
| TRANSLATIONAL_INITIATION | 25 | -1.788 | 0.103 |
| STRUCTURAL_MOLECULE_ACTIVITY | 180 | -1.773 | 0.114 |
| RNA_PROCESSING | 122 | -1.766 | 0.114 |
| NUCLEOLUS | 98 | -1.763 | 0.109 |
| LIGASE_ACTIVITY_FORMING_CARBON_OXYGEN_BONDS | 12 | -1.758 | 0.109 |
| NEGATIVE_REGULATION_OF_PROTEIN_METABOLIC_PROCESS | 39 | -1.757 | 0.105 |
| RIBONUCLEASE_ACTIVITY | 15 | -1.754 | 0.104 |
