# Supplementary material for: Molecular networks affected by neonatal microbial colonization in porcine jejunum, luminally perfused with enterotoxigenic Escherichia coli, F4ac fimbria or Lactobacillus amylovorus
Source: PLoS One. 2018 Aug 30;13(8):e0202160. doi: 10.1371/journal.pone.0202160 (PMC6116929; doi:10.1371/journal.pone.0202160)
Supplement: S4 Table — NES = normalized enrichment score; FDR = false discovery rate. (DOCX) [file pone.0202160.s006.docx]

**S4 Table.** **Ordered list of the first twenty groups of genes down-regulated in F4 treated loops, compared to CTRL loops (NES, normalized enrichment score; FDR, false discovery rate).**

| NAME | SIZE | NES | FDR q-val |
| --- | --- | --- | --- |
| RIBONUCLEOPROTEIN_COMPLEX_BIOGENESIS_AND_ASSEMBLY | 58 | -2.343 | 0.000 |
| TRANSLATION | 131 | -2.163 | 0.003 |
| TRANSLATION_FACTOR_ACTIVITY_NUCLEIC_ACID_BINDING | 27 | -2.116 | 0.003 |
| TRANSLATION_REGULATOR_ACTIVITY | 28 | -2.104 | 0.003 |
| CELLULAR_BIOSYNTHETIC_PROCESS | 241 | -2.087 | 0.004 |
| NEGATIVE_REGULATION_OF_CELLULAR_PROTEIN_METABOLIC_PROCESS | 36 | -2.073 | 0.004 |
| NEGATIVE_REGULATION_OF_PROTEIN_METABOLIC_PROCESS | 39 | -2.068 | 0.004 |
| RNA_PROCESSING | 122 | -2.046 | 0.005 |
| PROTEIN_RNA_COMPLEX_ASSEMBLY | 43 | -2.022 | 0.005 |
| NUCLEOLUS | 98 | -2.020 | 0.005 |
| STRUCTURAL_CONSTITUENT_OF_RIBOSOME | 56 | -1.997 | 0.006 |
| TRANSLATIONAL_INITIATION | 25 | -1.997 | 0.006 |
| REGULATION_OF_CELLULAR_PROTEIN_METABOLIC_PROCESS | 121 | -1.996 | 0.005 |
| TRNA_METABOLIC_PROCESS | 16 | -1.991 | 0.006 |
| TRANSLATION_INITIATION_FACTOR_ACTIVITY | 17 | -1.966 | 0.009 |
| REGULATION_OF_TRANSLATIONAL_INITIATION | 19 | -1.948 | 0.011 |
| REGULATION_OF_TRANSLATION | 69 | -1.948 | 0.010 |
| RIBONUCLEOPROTEIN_COMPLEX | 101 | -1.926 | 0.013 |
| REGULATION_OF_PROTEIN_METABOLIC_PROCESS | 131 | -1.899 | 0.017 |
| NEGATIVE_REGULATION_OF_BIOSYNTHETIC_PROCESS | 23 | -1.844 | 0.033 |
