# Supplementary material for: Molecular networks affected by neonatal microbial colonization in porcine jejunum, luminally perfused with enterotoxigenic Escherichia coli, F4ac fimbria or Lactobacillus amylovorus
Source: PLoS One. 2018 Aug 30;13(8):e0202160. doi: 10.1371/journal.pone.0202160 (PMC6116929; doi:10.1371/journal.pone.0202160)
Supplement: S5 Table — n.a.: not assigned. (DOCX) [file pone.0202160.s007.docx]

**S5 Table.** **Statistically significant genes (false discovery rate, P<0.05) for the pairwise contrast ETEC vs. CTRL, ordered for fold change.** n.a.: not assigned.

| Transcript Cluster ID | ETEC, Signal (log2) | CTRL, Signal (log2) | Fold Change (linear) | FDR p-value (ETEC vs. CONTR) | Gene Symbol | Description |
| --- | --- | --- | --- | --- | --- | --- |
| 15334440 | 8.74 | 3.69 | 33.03 | 0.0002 | NOX1 | NADPH oxidase 1 |
| 15212868 | 8.94 | 4.47 | 22.1 | 0.0005 | NOS2 | nitric oxide synthase 2, inducible |
| 15293045 | 7.38 | 2.96 | 21.38 | 0.0042 | IL22 | interleukin 22 |
| 15330106 | 7.35 | 3.12 | 18.81 | 0.0005 | MMP12 | matrix metallopeptidase 12 (macrophage elastase) |
| 15346507 | 10.59 | 6.36 | 18.71 | 0.0001 | n.a. |  |
| 15323385 | 7.55 | 3.48 | 16.78 | 0.0007 | CXCL11 | chemokine (C-X-C motif) ligand 11 |
| 15323375 | 7.24 | 3.55 | 12.9 | 0.0032 | CXCL9 | chemokine (C-X-C motif) ligand 9 |
| 15275761 | 9.59 | 5.96 | 12.34 | 0.0007 | REG3G | regenerating islet-derived 3 gamma |
| 15323380 | 9.73 | 6.12 | 12.21 | 0.0011 | CXCL10 | chemokine (C-X-C motif) ligand 10 |
| 15197302 | 7.33 | 3.97 | 10.29 | 0.0010 | CD274 | CD274 molecule |
| 15298968 | 7.39 | 4.22 | 9.03 | 0.0022 | PLA2G2D | phospholipase A2, group IID |
| 15286334 | 7.51 | 4.62 | 7.41 | 0.0002 | CHI3L2 | chitinase 3-like 2 isoform A |
| 15186351 | 7.57 | 4.82 | 6.73 | 0.0005 | DUOX2 | dual oxidase 2; dual oxidase 1 |
| 15295924 | 6.55 | 4.11 | 5.43 | 0.0002 | HP | haptoglobin |
| 15186344 | 7.15 | 4.73 | 5.37 | 0.0007 | DUOXA2 | dual oxidase maturation factor 2 |
| 15286881 | 7.79 | 5.51 | 4.87 | 0.0005 | GBP2 | guanylate binding protein 2, interferon-inducible |
| 15282309 | 9.23 | 7 | 4.7 | 0.0003 | GBP1 | guanylate binding protein 1, interferon-inducible |
| 15318201 | 10.61 | 8.42 | 4.56 | 0.0009 | GPX2 | glutathione peroxidase 2 (gastrointestinal) |
| 15350217 | 7.07 | 4.88 | 4.56 | 0.0013 | n.a. |  |
| 15341079 | 8.44 | 6.25 | 4.54 | 0.0016 | SOCS1 | suppressor of cytokine signaling 1 |
| 15323288 | 7.4 | 5.34 | 4.17 | 0.0132 | AMCF-II | alveolar macrophage-derived chemotactic factor-II |
| 15351241 | 5.78 | 3.75 | 4.06 | 0.0006 | CYP8B1 | cytochrome P450, family 8, subfamily B, polypeptide 1 |
| 15275023 | 6.3 | 4.29 | 4.02 | 0.0156 | IL1A | interleukin 1, alpha |
| 15200534 | 6.94 | 5.03 | 3.75 | 0.0004 | SLC28A3 | solute carrier family 28 (sodium-coupled nucleoside transporter), member 3 |
| 15194839 | 4.5 | 2.62 | 3.7 | 0.0051 | n.a. |  |
| 15218476 | 7.66 | 5.79 | 3.66 | 0.0005 | ABCC13 | ATP-Binding Cassette, Sub-Family C (CFTR/MRP), Member 13 |
| 15332374 | 5.41 | 3.55 | 3.63 | 0.0004 | n.a. |  |
| 15291749 | 7.77 | 5.93 | 3.58 | 0.0376 | RND1 | Rho Family GTPase 1 |
| 15215850 | 6.41 | 4.64 | 3.42 | 0.0003 | ITIH4 | inter-alpha-trypsin inhibitor heavy chain family, member 4 |
| 15281489 | 10.8 | 9.04 | 3.4 | 0.0002 | BCL2L15 | bcl-2-like protein 15 |
| 15292014 | 9 | 7.25 | 3.36 | 0.0034 | CXCL2 | chemokine (C-X-C motif) ligand 2 |
| 15323275 | 9 | 7.25 | 3.36 | 0.0034 | CXCL2 | chemokine (C-X-C motif) ligand 2 |
| 15349029 | 6.81 | 5.12 | 3.23 | 0.0020 | SLC7A11 | Solute Carrier Family 7 (Anionic Amino Acid Transporter Light Chain, Xc- System), Member 11 |
| 15199278 | 5.3 | 3.63 | 3.19 | 0.0177 | ASS1 | argininosuccinate synthase 1 |
| 15306957 | 9.19 | 7.52 | 3.18 | 0.0002 | TSPAN1 | tetraspanin 1 |
| 15320849 | 10.69 | 9.04 | 3.14 | 0.0034 | IL8 | interleukin 8 |
| 15212659 | 7.14 | 5.53 | 3.06 | 0.0043 | CCL8 | chemokine (C-C motif) ligand 8 |
| 15330095 | 9.75 | 8.14 | 3.05 | 0.0044 | MMP3 | matrix metallopeptidase 3 (stromelysin 1, progelatinase) |
| 15232454 | 5.37 | 3.8 | 2.97 | 0.0027 | DNAJC12 | dnaJ homolog subfamily C member 12 |
| 15351217 | 5.65 | 4.14 | 2.85 | 0.0100 | LTF | Lactotransferrin |
| 15183956 | 7.07 | 5.56 | 2.83 | 0.0218 | ARG1 | arginase, liver |
| 15343201 | 6.83 | 5.35 | 2.79 | 0.0014 | GBP5 | guanylate binding protein 5 |
| 15194363 | 10.6 | 9.13 | 2.76 | 0.0007 | GCNT3 | beta-1,3-galactosyl-O-glycosyl-glycoprotein beta-1,6-N-acetylglucosaminyltransferase 3 |
| 15273418 | 10.6 | 9.13 | 2.76 | 0.0007 | GCNT3 | beta-1,3-galactosyl-O-glycosyl-glycoprotein beta-1,6-N-acetylglucosaminyltransferase 3 |
| 15320088 | 6.97 | 5.51 | 2.76 | 0.0012 | C4orf19 | Chromosome 4 Open Reading Frame 19 |
| 15300998 | 9.56 | 8.12 | 2.71 | 0.0002 | TSPAN1 | tetraspanin 1 |
| 15302160 | 4.89 | 3.45 | 2.7 | 0.0023 | CCL22 | chemokine (C-C motif) ligand 22 |
| 15270826 | 11.59 | 10.19 | 2.65 | 0.0034 | REG3G | regenerating islet-derived 3 gamma |
| 15275764 | 11.59 | 10.19 | 2.65 | 0.0034 | REG3G | regenerating islet-derived 3 gamma |
| 15215050 | 8.47 | 7.09 | 2.61 | 0.0001 | HIGD1A | HIG1 Hypoxia Inducible Domain Family, Member 1A |
| 15262875 | 7.61 | 6.26 | 2.56 | 0.0002 | NUCB2 | nucleobindin 2 |
| 15287934 | 6.68 | 5.33 | 2.56 | 0.0045 | CXCL2 | chemokine (C-X-C motif) ligand 2 |
| 15330595 | 9.26 | 7.93 | 2.52 | 0.0003 | HYOU1 | hypoxia up-regulated 1 |
| 15185283 | 6.43 | 5.11 | 2.49 | 0.0177 | LIPG | lipase, endothelial |
| 15324478 | 6.96 | 5.65 | 2.47 | 0.0011 | SPP1 | secreted phosphoprotein 1 |
| 15349271 | 4.89 | 3.6 | 2.45 | 0.0108 | SLC10A2 | Solute Carrier Family 10 (Sodium/Bile Acid Cotransporter), Member 2 |
| 15330117 | 7.43 | 6.15 | 2.43 | 0.0184 | MMP13 | matrix metalloproteinase 13 precursor |
| 15282291 | 8.27 | 7.01 | 2.39 | 0.0027 | GBP4 | guanylate binding protein 4 |
| 15340485 | 7.02 | 5.77 | 2.38 | 0.0003 | n.a. |  |
| 15331406 | 7.21 | 5.96 | 2.38 | 0.0005 | ASNS | asparagine synthetase (glutamine-hydrolyzing) |
| 15231692 | 6.77 | 5.53 | 2.37 | 0.0002 | SDF2L1 | stromal cell-derived factor 2 |
| 15319270 | 9.81 | 8.56 | 2.37 | 0.0017 | WARS | Tryptophanyl-TRNA Synthetase |
| 15317865 | 10.05 | 8.82 | 2.34 | 0.0002 | PNP | purine nucleoside phosphorylase |
| 15339779 | 6.98 | 5.77 | 2.33 | 0.0323 | BATF2 | Basic Leucine Zipper Transcription Factor,2 |
| 15328878 | 8.08 | 6.87 | 2.32 | 0.0047 | n.a. |  |
| 15212042 | 8.42 | 7.22 | 2.29 | 0.0005 | ABCC3 | ATP-binding cassette, sub-family C (CFTR/MRP), member 3 |
| 15244402 | 7.6 | 6.41 | 2.29 | 0.0017 | CCL28 | chemokine (C-C motif) ligand 28 |
| 15308169 | 5.59 | 4.4 | 2.29 | 0.0070 | RNF39 | ring finger protein 39 |
| 15289424 | 5.91 | 4.71 | 2.29 | 0.0206 | CD69 | CD69 molecule |
| 15217790 | 4.37 | 3.19 | 2.27 | 0.0111 | MUC4 | mucin 4, cell surface associated |
| 15216530 | 6.29 | 5.12 | 2.26 | 0.0014 | PPARG | peroxisome proliferator-activated receptor gamma |
| 15220196 | 8.79 | 7.63 | 2.24 | 0.0002 | MANF | mesencephalic astrocyte-derived neurotrophic factor |
| 15323982 | 4.94 | 3.78 | 2.24 | 0.0279 | TNIP3 | TNFAIP3 Interacting Protein 3 |
| 15290930 | 7.84 | 6.69 | 2.23 | 0.0011 | CRELD2 | Cysteine-Rich With EGF-Like Domains 2 |
| 15349593 | 5.6 | 4.45 | 2.22 | 0.0007 | n.a. |  |
| 15197296 | 6.12 | 4.98 | 2.21 | 0.0003 | PDCD1LG2 | programmed cell death 1 ligand 2 |
| 15217560 | 9.46 | 8.32 | 2.2 | 0.0001 | DNAJB11 | DnaJ (Hsp40) homolog, subfamily B, member 11 |
| 15227319 | 7.43 | 6.29 | 2.2 | 0.0013 | TMEM72 | transmembrane protein 72 |
| 15233271 | 7.47 | 6.35 | 2.18 | 0.0021 | TMEM72 | transmembrane protein 72 |
| 15293768 | 7.44 | 6.32 | 2.17 | 0.0002 | CLEC2B | C-type lectin domain family 2, member B |
| 15222185 | 9.1 | 7.99 | 2.17 | 0.0007 | SLC51A | Solute Carrier Family 51, Alpha Subunit |
| 15271131 | 10.62 | 9.53 | 2.14 | 0.0002 | GFPT1 | glutamine--fructose-6-phosphate transaminase 1 |
| 15328872 | 8.12 | 7.02 | 2.14 | 0.0035 | n.a. |  |
| 15215609 | 8.22 | 7.12 | 2.14 | 0.0147 | CISH | Cytokine Inducible SH2-Containing Protein |
| 15339269 | 8.18 | 7.08 | 2.14 | 0.0253 | SOCS3 | suppressor of cytokine signaling 3 |
| 15248074 | 3.87 | 2.78 | 2.12 | 0.0096 | IDO1 | indoleamine 2,3-dioxygenase 1 |
| 15280666 | 5.79 | 4.71 | 2.12 | 0.0719 | S100A9 | S100 calcium binding protein A9 |
| 15223090 | 8.23 | 7.15 | 2.11 | 0.0008 | LPL | lipoprotein lipase |
| 15349211 | 5.35 | 4.28 | 2.1 | 0.0019 | n.a. |  |
| 15260296 | 7.38 | 6.31 | 2.1 | 0.0032 | IFITM1 | interferon induced transmembrane protein 1 |
| 15196550 | 5.73 | 4.67 | 2.09 | 0.0005 | GNPNAT1 | glucosamine-phosphate N-acetyltransferase 1 |
| 15349449 | 4.55 | 3.49 | 2.09 | 0.0005 | n.a. |  |
| 15315177 | 9.42 | 8.37 | 2.08 | 0.0012 | TAP1 | transporter 1, ATP-binding cassette, sub-family B (MDR/TAP) |
| 15315205 | 9.42 | 8.37 | 2.08 | 0.0012 | TAP1 | transporter 1, ATP-binding cassette, sub-family B (MDR/TAP) |
| 15270156 | 5.5 | 4.45 | 2.08 | 0.0080 | n.a. | E3 ubiquitin-protein ligase NEURL3-like |
| 15339507 | 5.47 | 4.42 | 2.08 | 0.0156 | SLC6A14 | Solute Carrier Family 6 (Amino Acid Transporter), Member 14 |
| 15323295 | 4.6 | 3.55 | 2.08 | 0.0499 | AREG | amphiregulin |
| 15239264 | 7.59 | 6.55 | 2.07 | 0.0012 | NRG1 | Neuregulin 1 |
| 15276167 | 7.5 | 6.45 | 2.07 | 0.0143 | PLEK | pleckstrin |
| 15351099 | 6.37 | 5.33 | 2.06 | 0.0010 | IL2RA | Interleukin 2 Receptor, Alpha |
| 15261838 | 6.85 | 5.81 | 2.06 | 0.0023 | FAM111A | Family With Sequence Similarity 111, Member A |
| 15350047 | 5.35 | 4.32 | 2.05 | 0.0010 | n.a. |  |
| 15324786 | 8.6 | 7.57 | 2.04 | 0.0025 | n.a. |  |
| 15278850 | 7.62 | 6.6 | 2.03 | 0.0008 | TRPA1 | transient receptor potential cation channel, subfamily A, member 1 |
| 15227478 | 6.65 | 5.63 | 2.03 | 0.0096 | IFIT2 | Interferon-Induced Protein With Tetratricopeptide Repeats 2 |
| 15261503 | 5.73 | 4.7 | 2.03 | 0.0220 | PHEROC | pheromaxein C subunit |
| 15233495 | 4.54 | 3.52 | 2.02 | 0.0015 | RBP4 | retinol binding protein 4, plasma |
| 15350413 | 7.16 | 6.15 | 2.02 | 0.0112 | n.a. |  |
| 15220015 | 6.7 | 5.69 | 2.01 | 0.0008 | GMPPB | GDP-mannose pyrophosphorylase B |
| 15251041 | 5.99 | 4.98 | 2.01 | 0.0013 | TFEC | Transcription Factor EC |
| 15233743 | 6.78 | 5.77 | 2.01 | 0.0057 | AVPI1 | vasopressin-induced protein, 32kDa |
| 15270952 | 7 | 8.01 | -2.01 | 0.0002 | RTKN | rhotekin |
| 15227377 | 8.73 | 9.74 | -2.01 | 0.0002 | A1CF | APOBEC1 complementation factor |
| 15307699 | 7.6 | 8.6 | -2.01 | 0.0003 | MYLIP | myosin regulatory light chain interacting protein |
| 15333687 | 9.2 | 10.2 | -2.01 | 0.0006 | MAOA | monoamine oxidase A |
| 15276624 | 9.15 | 10.16 | -2.01 | 0.0008 | ABCG5 | ATP-binding cassette, sub-family G (WHITE), member 5 |
| 15244049 | 8.51 | 9.52 | -2.02 | 0.0001 | PRLR | prolactin receptor |
| 15343395 | 3.45 | 4.46 | -2.02 | 0.0003 | PTPRR | Protein Tyrosine Phosphatase, Receptor Type, R |
| 15202719 | 8.5 | 9.51 | -2.02 | 0.0012 | PTER | phosphotriesterase related |
| 15248635 | 7.39 | 8.41 | -2.03 | 0.0002 | ATRN | attractin |
| 15270596 | 5.56 | 6.58 | -2.03 | 0.0002 | CD8B | CD8B |
| 15235244 | 7.85 | 8.87 | -2.03 | 0.0003 | SLC35F5 | solute carrier family 35, member F5 |
| 15344561 | 7.65 | 8.68 | -2.03 | 0.0007 | n.a. |  |
| 15275925 | 6.28 | 7.3 | -2.03 | 0.0024 | NAT8 | N-acetyltransferase 8B |
| 15278143 | 8.11 | 9.13 | -2.04 | 0.0006 | ENPP2 | ectonucleotide pyrophosphatase/phosphodiesterase 2 |
| 15271170 | 6.78 | 7.81 | -2.04 | 0.0006 | FBXO48 | F-box only protein 48 |
| 15341163 | 8.98 | 10 | -2.04 | 0.0009 | n.a. |  |
| 15235051 | 7.38 | 8.41 | -2.04 | 0.0027 | PRAP1 | proline-rich acidic protein 1-like |
| 15219248 | 7.87 | 8.9 | -2.05 | 0.0002 | CTDSPL | CTD (Carboxy-Terminal Domain, RNA Polymerase II, Polypeptide A) Small Phosphatase-Like |
| 15293412 | 8.03 | 9.07 | -2.05 | 0.0005 | ITPR2 | Inositol 1,4,5-Trisphosphate Receptor, Type 2 |
| 15219047 | 6.14 | 7.18 | -2.05 | 0.0005 | THRB | thyroid hormone receptor beta 1 (c-erbA-beta 1) |
| 15349249 | 6.59 | 7.62 | -2.05 | 0.0008 | n.a. |  |
| 15217243 | 4.95 | 5.98 | -2.05 | 0.0014 | SERPINI1 | neuroserpin |
| 15346441 | 6.2 | 7.25 | -2.06 | 0.0005 | n.a. |  |
| 15262467 | 10.55 | 11.59 | -2.06 | 0.0006 | CAT | catalase |
| 15350529 | 5.45 | 6.49 | -2.06 | 0.0013 | CYP2C36 | cytochrome P450, family 2, subfamily C, polypeptide 36 |
| 15313810 | 8.1 | 9.15 | -2.07 | 0.0002 | SLC28A1 | Na/nucleoside cotransporter |
| 15275195 | 7.26 | 8.31 | -2.07 | 0.0002 | ZNF514 | zinc finger protein 514; zinc finger protein 514-like |
| 15346399 | 3.79 | 4.84 | -2.07 | 0.0008 | n.a. |  |
| 15271902 | 7.75 | 8.81 | -2.08 | 0.0004 | ATL2 | Atlastin GTPase 2 |
| 15191849 | 5.22 | 6.28 | -2.08 | 0.0005 | FRMD1 | FERM domain-containing protein 1 |
| 15194682 | 7.84 | 8.89 | -2.08 | 0.0014 | SLC27A2 | solute carrier family 27 (fatty acid transporter), member 2 |
| 15225453 | 10.19 | 11.25 | -2.08 | 0.0018 | SLC5A1 | solute carrier family 5 (sodium/glucose cotransporter), member 1 |
| 15274465 | 7.77 | 8.84 | -2.09 | 0.0001 | HMOX2 | heme oxygenase 2 |
| 15212033 | 7.53 | 8.6 | -2.09 | 0.0004 | n.a. |  |
| 15310214 | 5.53 | 6.59 | -2.09 | 0.0017 | CYP39A1 | cytochrome P450, family 39, subfamily A, polypeptide 1 |
| 15313262 | 5.65 | 6.71 | -2.09 | 0.0018 | SLC24A4 | Solute Carrier Family 24 (Sodium/Potassium/Calcium Exchanger), Member 4 |
| 15299714 | 6.29 | 7.36 | -2.09 | 0.0018 | n.a. |  |
| 15330586 | 8.19 | 9.26 | -2.1 | 0.0003 | SLC37A4 | solute carrier family 37 (glucose-6-phosphate transporter), member 4 |
| 15186116 | 7.16 | 8.23 | -2.1 | 0.0012 | SLC27A2 | solute carrier family 27 (fatty acid transporter), member 2 |
| 15306909 | 6.06 | 7.14 | -2.11 | 0.0005 | FAAH | fatty acid amide hydrolase |
| 15277376 | 5.9 | 6.98 | -2.12 | 0.0001 | RNF144A | Ring Finger Protein 144A |
| 15185449 | 6.74 | 7.83 | -2.12 | 0.0002 | SPG21 | spastic paraplegia 21 (autosomal recessive, Mast syndrome) |
| 15206066 | 5.89 | 6.98 | -2.13 | 0.0001 | SLC26A11 | solute carrier family 26, member 11 |
| 15341935 | 4.57 | 5.68 | -2.15 | 0.0002 | PCSK4 | Proprotein Convertase Subtilisin/Kexin Type 4 |
| 15239851 | 8.25 | 9.35 | -2.15 | 0.0011 | CYBRD1 | cytochrome b reductase 1 |
| 15223489 | 6.2 | 7.31 | -2.15 | 0.0021 | EPHX2 | epoxide hydrolase 2, cytoplasmic |
| 15254088 | 5.12 | 6.24 | -2.16 | 0.0001 | SLC25A45 | solute carrier family 25 member 45 |
| 15211823 | 6.22 | 7.34 | -2.16 | 0.0002 | ERBB2 | receptor tyrosine-protein kinase erbB-2 |
| 15346443 | 7.76 | 8.87 | -2.16 | 0.0004 | GDPD1 | glycerophosphodiester phosphodiesterase domain containing 1 |
| 15302581 | 4.97 | 6.08 | -2.16 | 0.0006 | SYNE4 | Spectrin Repeat Containing, Nuclear Envelope Family Member 4 |
| 15341165 | 8.97 | 10.08 | -2.16 | 0.0008 | SGK1 | Serum/Glucocorticoid Regulated Kinase 1 |
| 15287255 | 5.24 | 6.35 | -2.17 | 0.0002 | PMM1 | phosphomannomutase 1 |
| 15311892 | 6.9 | 8.03 | -2.18 | 0.0002 | ABHD4 | abhydrolase domain containing 4 |
| 15321734 | 5.33 | 6.45 | -2.18 | 0.0002 | GPRIN3 | G protein-regulated inducer of neurite outgrowth 3 |
| 15191870 | 6.96 | 8.08 | -2.18 | 0.0002 | UNC93A | protein unc-93 homolog A |
| 15292977 | 5.74 | 6.87 | -2.18 | 0.0003 | TBC1D30 | TBC1 Domain Family, Member 30 |
| 15242381 | 7.51 | 8.63 | -2.18 | 0.0003 | ANKH | ANKH Inorganic Pyrophosphate Transport Regulator |
| 15205575 | 8.79 | 9.93 | -2.19 | 0.0003 | SLC15A1 | solute carrier family 15 (oligopeptide transporter), member 1 |
| 15208088 | 6.05 | 7.19 | -2.19 | 0.0003 | ACSF2 | Acyl-CoA Synthetase Family Member 2 |
| 15323474 | 7.79 | 8.92 | -2.19 | 0.0011 | LRAT | lecithin retinol acyltransferase (phosphatidylcholine--retinol O-acyltransferase) |
| 15325951 | 10.17 | 11.3 | -2.19 | 0.0056 | APOC3 | apolipoprotein C-III |
| 15305493 | 5.58 | 6.72 | -2.2 | 0.0002 | SESN2 | sestrin-2 |
| 15239349 | 3.25 | 4.39 | -2.2 | 0.0200 | n.a. |  |
| 15239351 | 3.25 | 4.39 | -2.2 | 0.0200 | n.a. |  |
| 15206144 | 4.64 | 5.78 | -2.21 | 0.0002 | ST6GALNAC1 | ST6 (alpha-N-acetyl-neuraminyl-2,3-beta-galactosyl-1,3)-N-acetylgalactosaminide alpha-2,6-sialyltransferase 1 |
| 15239492 | 7.08 | 8.22 | -2.21 | 0.0003 | CD302 | CD302 molecule |
| 15316286 | 6.31 | 7.46 | -2.21 | 0.0007 | CYP39A1 | cytochrome P450, family 39, subfamily A, polypeptide 1 |
| 15250624 | 6.91 | 8.05 | -2.21 | 0.0008 | TMEM140 | transmembrane protein 140 |
| 15308597 | 5.07 | 6.21 | -2.21 | 0.0014 | APOM | apolipoprotein M |
| 15341167 | 9 | 10.15 | -2.22 | 0.0005 | SGK1 | Serum/Glucocorticoid Regulated Kinase 1 |
| 15296672 | 4.52 | 5.67 | -2.22 | 0.0008 | n.a. | nesprin-4-like |
| 15259447 | 6.79 | 7.95 | -2.22 | 0.0015 | LEAP2 | liver expressed antimicrobial peptide 2 |
| 15232678 | 6.43 | 7.59 | -2.23 | 0.0002 | PLA2G12B | phospholipase A2, group XIIB |
| 15271702 | 8.16 | 9.31 | -2.23 | 0.0006 | n.a. | ATP-binding cassette, sub-family G (WHITE), member 5 |
| 15346401 | 3.75 | 4.91 | -2.23 | 0.0020 | n.a. |  |
| 15226646 | 7.61 | 8.78 | -2.24 | 0.0001 | SGPL1 | sphingosine-1-phosphate lyase 1 |
| 15271772 | 7.25 | 8.42 | -2.25 | 0.0002 | HAAO | 3-hydroxyanthranilate 3,4-dioxygenase |
| 15239440 | 3.96 | 5.13 | -2.25 | 0.0002 | ACVR1C | activin A receptor, type IC |
| 15330204 | 7.66 | 8.83 | -2.25 | 0.0005 | EXPH5 | exophilin 5 |
| 15197472 | 4.25 | 5.42 | -2.25 | 0.0050 | C9orf71 | transmembrane protein C9orf71 |
| 15282529 | 5.41 | 6.58 | -2.26 | 0.0002 | GPT | Glutamic-Pyruvate Transaminase (Alanine Aminotransferase) |
| 15339353 | 9.77 | 10.95 | -2.26 | 0.0025 | CYP3A46 | cytochrome P450, family 3, subfamily A, polypeptide 46 |
| 15343435 | 7.09 | 8.27 | -2.26 | 0.0332 | - | Sulfotransferase |
| 15303191 | 6.58 | 7.78 | -2.28 | 0.0002 | CBLC | Cbl Proto-Oncogene C, E3 Ubiquitin Protein Ligase |
| 15197544 | 3.96 | 5.15 | -2.28 | 0.0005 | GDA | guanine deaminase |
| 15287465 | 8.02 | 9.21 | -2.29 | 0.0003 | BAIAP2L2 | BAI1-Associated Protein 2-Like 2 |
| 15338042 | 6.13 | 7.34 | -2.31 | 0.0001 | KLHL13 | kelch-like protein 13 |
| 15209008 | 6.48 | 7.69 | -2.31 | 0.0001 | PIPOX | peroxisomal sarcosine oxidase |
| 15314771 | 5.46 | 6.67 | -2.31 | 0.0001 | SFTA2 | surfactant-associated protein 2 |
| 15351239 | 9.66 | 10.87 | -2.31 | 0.0026 | CYP3A29 | cytochrome P450, family 3, subfamily A, polypeptide 29 |
| 15337135 | 4.49 | 5.72 | -2.35 | 0.0001 | EDA | ectodysplasin |
| 15339207 | 6.35 | 7.58 | -2.35 | 0.0007 | PHYH | Phytanoyl-CoA 2-Hydroxylase |
| 15192204 | 5.29 | 6.53 | -2.36 | 0.0005 | IYD | iodotyrosine deiodinase |
| 15200442 | 7.76 | 9 | -2.37 | 0.0002 | FBP2 | fructose-1,6-bisphosphatase 2 |
| 15318239 | 5.7 | 6.95 | -2.38 | 0.0003 | TMEM229B | transmembrane protein 229B |
| 15347235 | 6.27 | 7.52 | -2.39 | 0.0002 | n.a. |  |
| 15281134 | 7.79 | 9.05 | -2.39 | 0.0004 | PDZK1 | PDZ domain containing 1 |
| 15310241 | 9.25 | 10.52 | -2.41 | 0.0007 | MEP1A | meprin A, alpha (PABA peptide hydrolase) |
| 15325225 | 6.33 | 7.61 | -2.42 | 0.0002 | TSKU | Tsukushi, Small Leucine Rich Proteoglycan |
| 15270837 | 4.57 | 5.85 | -2.42 | 0.0002 | EVA1A | Eva-1 Homolog A (C. Elegans) |
| 15217968 | 6.26 | 7.54 | -2.43 | 0.0013 | n.a. |  |
| 15306590 | 4.18 | 5.47 | -2.44 | 0.0029 | CYP2J33 | cytochrome P450, family 2, subfamily J, polypeptide 34 |
| 15350051 | 7.34 | 8.64 | -2.46 | 0.0002 | n.a. |  |
| 15228243 | 8.33 | 9.63 | -2.46 | 0.0004 | AS3MT | Arsenite Methyltransferase |
| 15283677 | 6.37 | 7.67 | -2.46 | 0.0005 | CA13 | carbonic anhydrase 13 |
| 15348025 | 2.87 | 4.17 | -2.46 | 0.0014 | UPP2 | Uridine Phosphorylase 2 |
| 15346397 | 3.8 | 5.12 | -2.49 | 0.0015 | PHOSPHO1 | phosphatase, orphan 1 |
| 15231243 | 6.58 | 7.91 | -2.5 | 0.0001 | NIPSNAP1 | nipsnap homolog 1 |
| 15243844 | 6.74 | 8.07 | -2.51 | 0.0005 | FAM134B | Family With Sequence Similarity 13, Member B |
| 15225264 | 5.95 | 7.28 | -2.52 | 0.0005 | SEC14L2 | SEC14-like 2 (S. cerevisiae) |
| 15341419 | 6.49 | 7.83 | -2.53 | 0.0002 | n.a. |  |
| 15287475 | 7.07 | 8.42 | -2.54 | 0.0004 | BAIAP2L2 | brain-specific angiogenesis inhibitor 1-associated protein 2 |
| 15230062 | 9.29 | 10.64 | -2.55 | 0.0001 | SCARB1 | scavenger receptor class B, member 1 |
| 15268536 | 5.19 | 6.55 | -2.56 | 0.0015 | PSPH | phosphoserine phosphatase |
| 15239744 | 8.12 | 9.48 | -2.56 | 0.0019 | LASS6 | ceramide synthase 6 |
| 15333731 | 5.84 | 7.21 | -2.57 | 0.0002 | RGN | regucalcin (senescence marker protein-30) |
| 15322458 | 6.76 | 8.14 | -2.6 | 0.0003 | n.a. |  |
| 15200721 | 6.37 | 7.76 | -2.61 | 0.0005 | AQP7 | aquaporin 7 |
| 15235425 | 8.73 | 10.12 | -2.62 | 0.0004 | TMEM37 | Transmembrane Protein 37 |
| 15304798 | 4.32 | 5.72 | -2.64 | 0.0001 | ARHGEF19 | Rho guanine nucleotide exchange factor (GEF) 19 |
| 15325200 | 7.16 | 8.57 | -2.64 | 0.0002 | DGAT2 | diacylglycerol O-acyltransferase 2 |
| 15231335 | 7.02 | 8.43 | -2.65 | 0.0008 | GAL3ST1 | galactose-3-O-sulfotransferase 1 |
| 15191764 | 5.69 | 7.1 | -2.66 | 0.0002 | ENTPD8 | ectonucleoside triphosphate diphosphohydrolase 8-like |
| 15226165 | 8.15 | 9.56 | -2.67 | 0.0002 | ABCB10 | ATP-binding cassette sub-family B member 10, mitochondrial |
| 15227406 | 9.16 | 10.57 | -2.67 | 0.0002 | PAPSS2 | bifunctional 3'-phosphoadenosine 5'-phosphosulfate synthase 2 |
| 15343699 | 7.01 | 8.43 | -2.68 | 0.0002 | n.a. |  |
| 15339883 | 3.65 | 5.07 | -2.68 | 0.0003 | n.a. |  |
| 15277952 | 5.86 | 7.29 | -2.7 | 0.0001 | GSDMC | gasdermin C |
| 15324604 | 6.55 | 7.99 | -2.71 | 0.0007 | AGPAT9 | 1-acylglycerol-3-phosphate O-acyltransferase 9 |
| 15350123 | 6.91 | 8.35 | -2.72 | 0.0003 | n.a. |  |
| 15259387 | 4.77 | 6.22 | -2.72 | 0.0005 | SLC22A4 | solute carrier family 22 (organic cation/ergothioneine transporter), member 4 |
| 15351021 | 6.11 | 7.57 | -2.74 | 0.0002 | PPARGC-1 | Peroxisome Proliferator-Activated Receptor Gamma, Coactivator 1 Beta |
| 15253153 | 5.23 | 6.68 | -2.74 | 0.0004 | PRR15 | proline-rich protein 15 |
| 15340223 | 5.52 | 6.98 | -2.75 | 0.0002 | n.a. |  |
| 15222161 | 6.88 | 8.35 | -2.76 | 0.0001 | BDH1 | 3-hydroxybutyrate dehydrogenase, type 1 |
| 15330668 | 5.16 | 6.63 | -2.77 | 0.0003 | USP2 | Ubiquitin Specific Peptidase 2 |
| 15233812 | 8.29 | 9.77 | -2.79 | 0.0002 | GOT1 | glutamic-oxaloacetic transaminase 1, soluble (aspartate aminotransferase 1) |
| 15300077 | 4.21 | 5.69 | -2.79 | 0.0006 | DSC1 | desmocollin-1 |
| 15255264 | 9.87 | 11.35 | -2.79 | 0.0009 | SLC5A12 | sodium-coupled monocarboxylate transporter 2 |
| 15227776 | 7.78 | 9.26 | -2.79 | 0.0020 | CYP2C49 | cytochrome P450 2C49 |
| 15258495 | 6 | 7.49 | -2.8 | 0.0002 | PDE8B | Phosphodiesterase 8B |
| 15200860 | 3.87 | 5.37 | -2.83 | 0.0015 | CUBN | Cubilin |
| 15223879 | 8.38 | 9.89 | -2.85 | 0.0002 | SCARB1 | scavenger receptor class B, member 1 |
| 15215339 | 7.52 | 9.04 | -2.86 | 0.0006 | SLC26A6 | Solute Carrier Family 26 (Anion Exchanger), Member 6 |
| 15339127 | 7.13 | 8.65 | -2.86 | 0.0007 | SLC2A2 | Solute Carrier Family 2 (Facilitated Glucose Transporter), Member 2 |
| 15279189 | 6.8 | 8.31 | -2.86 | 0.0008 | SDR16C5 | short chain dehydrogenase/reductase family 16C, member 5 |
| 15200426 | 8.51 | 10.03 | -2.87 | 0.0002 | FBP1 | fructose-1,6-bisphosphatase 1 |
| 15342855 | 5.87 | 7.39 | -2.88 | 0.0004 | n.a. |  |
| 15279681 | 6.56 | 8.09 | -2.88 | 0.0006 | NR1I3 | constitutive androstane receptor |
| 15301496 | 7.38 | 8.91 | -2.89 | 0.0001 | HSD17B2 | hydroxysteroid (17-beta) dehydrogenase 2 |
| 15217665 | 6.83 | 8.36 | -2.89 | 0.0002 | n.a. | NON ABBASTANZA SICURA D-beta-hydroxybutyrate dehydrogenase, mitochondrial-like |
| 15283324 | 5.21 | 6.75 | -2.89 | 0.0004 | OSR2 | odd-skipped related 2 (Drosophila) |
| 15219306 | 7.08 | 8.61 | -2.89 | 0.0004 | SLC22A13 | solute carrier family 22 (organic anion transporter), member 13 |
| 15185592 | 4.73 | 6.27 | -2.9 | 0.0002 | CA12 | Carbonic Anhydrase XII |
| 15239920 | 7.93 | 9.48 | -2.92 | 0.0003 | GPR155 | G Protein-Coupled Receptor 155 |
| 15350851 | 6.01 | 7.57 | -2.95 | 0.0081 | GPRC5A | G Protein-Coupled Receptor, Class C, Group 5, Member A |
| 15207641 | 5.52 | 7.1 | -2.98 | 0.0001 | RAPGEFL1 | Rap Guanine Nucleotide Exchange Factor (GEF) |
| 15343673 | 6.95 | 8.53 | -2.99 | 0.0002 | n.a. |  |
| 15295852 | 5.82 | 7.41 | -3 | 0.0002 | n.a. | PH domain leucine-rich repeat-containing protein phosphatase 2 |
| 15340081 | 5.24 | 6.84 | -3.03 | 0.0001 | n.a. |  |
| 15184913 | 7.99 | 9.6 | -3.06 | 0.0001 | SLC16A10 | Monocarboxylate transporter 10 |
| 15310223 | 7.48 | 9.09 | -3.06 | 0.0002 | SLC25A27 | solute carrier family 25, member 27 |
| 15221126 | 7.2 | 8.81 | -3.06 | 0.0002 | ACAD11 | acyl-Coenzyme A dehydrogenase family, member 11 |
| 15323541 | 5.34 | 6.96 | -3.08 | 0.0001 | FHDC1 | FH2 domain-containing protein 1 |
| 15339455 | 8.23 | 9.86 | -3.09 | 0.0004 | CLDN15 |  |
| 15228046 | 3.71 | 5.37 | -3.17 | 0.0300 | n.a. |  |
| 15222300 | 8.19 | 9.86 | -3.19 | 0.0006 | PARP15 | poly (ADP-ribose) polymerase family, member 15 |
| 15306839 | 5.16 | 6.84 | -3.21 | 0.0001 | SLC5A9 | sodium/glucose cotransporter 4 |
| 15197175 | 5.35 | 7.04 | -3.22 | 0.0002 | PTPRQ | protein tyrosine phosphatase, receptor type, D |
| 15346469 | 3.41 | 5.1 | -3.24 | 0.0002 | n.a. |  |
| 15351067 | 5.77 | 7.47 | -3.25 | 0.0001 | DPEP1 | Dipeptidase 1 (renal) |
| 15213125 | 5.48 | 7.18 | -3.26 | 0.0001 | TMIGD1 | Transmembrane And Immunoglobulin Domain Containing 1 |
| 15202314 | 7.63 | 9.34 | -3.26 | 0.0002 | FBP1 | fructose-1,6-bisphosphatase 1 |
| 15211807 | 4.99 | 6.72 | -3.32 | 0.0001 | GRB7 | growth factor receptor-bound protein 7 |
| 15307644 | 7.01 | 8.74 | -3.32 | 0.0003 | ADTRP | Androgen-Dependent TFPI-Regulating Protein |
| 15241329 | 5.59 | 7.36 | -3.42 | 0.0003 | CYP27A1 | cytochrome P450, family 27, subfamily A, polypeptide 1 |
| 15239464 | 5.17 | 6.98 | -3.51 | 0.0029 | UPP2 |  |
| 15216842 | 6.34 | 8.19 | -3.6 | 0.0001 | ACAD11 | acyl-Coenzyme A dehydrogenase family, member 11 |
| 15247708 | 4.17 | 6.02 | -3.6 | 0.0002 | PCK1 | phosphoenolpyruvate carboxykinase 1 (soluble) |
| 15349819 | 6.93 | 8.81 | -3.69 | 0.0008 | n.a. |  |
| 15265769 | 5.07 | 6.95 | -3.7 | 0.0004 | GCNT4 | glucosaminyl (N-acetyl) transferase 4, core 2 |
| 15184393 | 4.97 | 6.86 | -3.71 | 0.0004 | NT5E | 5 nucleotidase, ecto |
| 15208628 | 3.1 | 5.05 | -3.87 | 0.0007 | C17ORF78 | Chromosome 17 Open Reading Frame 78 |
| 15298397 | 5.64 | 7.6 | -3.89 | 0.0001 | SLC2A7 | solute carrier family 2 (facilitated glucose transporter), member 7 |
| 15302291 | 6.38 | 8.35 | -3.91 | 0.0001 | GPT2 | glutamic pyruvate transaminase (alanine aminotransferase) 2 |
| 15231468 | 8.64 | 10.63 | -3.97 | 0.0010 | SLC5A4 | solute carrier family 5 (low affinity glucose cotransporter), member 4 |
| 15285389 | 8.1 | 10.11 | -4.01 | 0.0009 | AQP10 | aquaporin 10 |
| 15272121 | 5.03 | 7.05 | -4.07 | 0.0006 | n.a. |  |
| 15209321 | 5.47 | 7.52 | -4.14 | 0.0002 | ASPA | aspartoacylase |
| 15222738 | 6.55 | 8.6 | -4.14 | 0.0025 | PRSS7 | transmembrane protease, serine 15 |
| 15235235 | 4.67 | 6.73 | -4.16 | 0.0001 | NCKAP5 | NCK-Associated Protein 5 |
| 15345959 | 3.85 | 5.92 | -4.18 | 0.0002 | CUBN | Cubilin |
| 15188896 | 5.49 | 7.58 | -4.25 | 0.0003 | n.a. |  |
| 15184389 | 6.28 | 8.37 | -4.26 | 0.0002 | NT5E | 5 nucleotidase, ecto |
| 15233325 | 7.95 | 10.05 | -4.28 | 0.0006 | ASAH2 | N-acylsphingosine amidohydrolase (non-lysosomal ceramidase) 2 |
| 15349869 | 4.02 | 6.16 | -4.4 | 0.0006 | n.a. |  |
| 15238132 | 7.41 | 9.61 | -4.61 | 0.0002 | KCNJ13 | potassium inwardly-rectifying channel, subfamily J, member 13 |
| 15347809 | 6.82 | 9.05 | -4.67 | 0.0008 | n.a. |  |
| 15202657 | 3.64 | 5.88 | -4.71 | 0.0003 | CUBN | Cubilin |
| 15349435 | 6.24 | 8.47 | -4.71 | 0.0003 | n.a. |  |
| 15243531 | 7.29 | 9.52 | -4.72 | 0.0001 | FAXDC2 | Fatty Acid Hydroxylase Domain Containing 2 |
| 15347095 | 3.69 | 6.1 | -5.31 | 0.0002 | ZBBX | Zinc Finger, B-Box Domain Containing |
| 15213111 | 7 | 9.42 | -5.33 | 0.0002 | SLC6A4 | Solute Carrier Family 6 (Neurotransmitter Transporter), Member 4 |
| 15183888 | 2.48 | 4.93 | -5.45 | 0.0006 | n.a. |  |
| 15343349 | 3.83 | 6.29 | -5.5 | 0.0002 | HSD17B6 | Hydroxysteroid (17-Beta) Dehydrogenase 6 |
| 15265960 | 5.7 | 8.19 | -5.62 | 0.0006 | ACOT12 | acyl-CoA thioesterase 12 |
| 15262622 | 6.51 | 9.02 | -5.68 | 0.0003 | n.a. |  |
| 15343347 | 3.82 | 6.39 | -5.96 | 0.0002 | n.a. |  |
| 15273933 | 6.52 | 9.11 | -6.03 | 0.0021 | AQP8 | aquaporin 8 |
| 15348027 | 4.19 | 6.79 | -6.05 | 0.0029 | n.a. |  |
| 15339205 | 8.47 | 11.13 | -6.36 | 0.0017 | G6PC | Glucose-6-Phosphatase, Catalytic Subunit |
| 15339363 | 5.5 | 8.18 | -6.41 | 0.0002 | BCO1 | Beta-Carotene Oxygenase 1 |
| 15210345 | 7.69 | 10.49 | -6.96 | 0.0008 | ENPP7 | ectonucleotide pyrophosphatase/phosphodiesterase 7 |
| 15247697 | 6.47 | 9.31 | -7.16 | 0.0004 | PCK1 | phosphoenolpyruvate carboxykinase 1 (soluble) |
| 15311843 | 6.2 | 9.21 | -8.09 | 0.0001 | SLC7A8 | Solute Carrier Family 7 (Amino Acid Transporter Light Chain, L System), Member 8 |
| 15183880 | 6.36 | 9.64 | -9.72 | 0.0001 | VNN1 | vanin 1 |
| 15250897 | 5.78 | 9.19 | -10.64 | 0.0009 | SLC13A1 | solute carrier family 13 (sodium/sulfate symporters), member 1 |
