# Supplementary material for: Molecular networks affected by neonatal microbial colonization in porcine jejunum, luminally perfused with enterotoxigenic Escherichia coli, F4ac fimbria or Lactobacillus amylovorus
Source: PLoS One. 2018 Aug 30;13(8):e0202160. doi: 10.1371/journal.pone.0202160 (PMC6116929; doi:10.1371/journal.pone.0202160)
Supplement: S6 Table — n.a.: not assigned. (DOCX) [file pone.0202160.s008.docx]

**S6 Table.** **Statistically significant genes (false discovery rate, P<0.05) for the pairwise contrast LAM vs. CTRL, ordered for fold change.** n.a.: not assigned.

| Transcript Cluster ID | ETEC, Signal (log2) | CTRL, Signal (log2) | Fold Change (linear) | FDR p-value (ETEC vs. CONTR) | Gene Symbol | Description |
| --- | --- | --- | --- | --- | --- | --- |
| 15237491 | 8.15 | 3.45 | 25.99 | 0.0030 | CPO | carboxypeptidase O |
| 15202657 | 10.11 | 5.88 | 18.73 | 0.0022 | CUBN | Cubilin |
| 15339349 | 10 | 6.92 | 8.5 | 0.0006 | SLC26A3 | Solute Carrier Family 26 (Anion Exchanger), Member 3 |
| 15276954 | 8.13 | 5.41 | 6.59 | 0.0013 | PLB1 | phospholipase B1 |
| 15213125 | 9.47 | 7.18 | 4.86 | 0.0004 | TMIGD1 | Transmembrane And Immunoglobulin Domain Containing 1 |
| 15197361 | 10.49 | 9.28 | 3.9 | 0.0005 | SLC1A1 | solute carrier family 1 (neuronal/epithelial high affinity glutamate transporter, system Xag), member 1 |
| 15283677 | 9.56 | 7.67 | 3.8 | 0.0005 | CA13 | carbonic anhydrase 13 |
| 15208561 | 7.7 | 5.77 | 3.8 | 0.0005 | CA4 | carbonic anhydrase IV |
| 15210657 | 8.11 | 6.18 | 3.8 | 0.0014 | OTOP2 | otopetrin-2 |
| 15294332 | 7.38 | 5.48 | 3.74 | 0.0004 | CPNE8 | copine VIII |
| 15212503 | 7.7 | 5.77 | 3.71 | 0.0005 | CA4 | carbonic anhydrase IV |
| 15243024 | 5.98 | 4.34 | 3.49 | 0.0004 | RNF180 | ring finger protein 180 |
| 15351067 | 9.19 | 7.47 | 3.29 | 0.0019 | DPEP1 | Dipeptidase 1 (Renal) |
| 15343511 | 7.97 | 6.17 | 3.11 | 0.0004 | CPNE8 | copine VIII |
| 15197472 | 6.87 | 5.42 | 2.74 | 0.0008 | TMEM252 | Transmembrane Protein 252 |
| 15233419 | 6.35 | 4.96 | 2.63 | 0.0069 | PPP1R3C | protein phosphatase 1 regulatory subunit 3C-like |
| 15186351 | 6.13 | 4.82 | 2.49 | 0.0018 | DUOX2 | dual oxidase 2 |
| 15350677 | 5.2 | 3.89 | 2.48 | 0.0041 | n.a. |  |
| 15227049 | 4.85 | 3.56 | 2.44 | 0.0029 | C10orf99 | chromosome 10 open reading frame 99 |
| 15250897 | 10.47 | 9.19 | 2.42 | 0.0312 | SLC13A1 | solute carrier family 13 (sodium/sulfate symporters), member 1 |
| 15301752 | 8.25 | 7 | 2.37 | 0.0006 | CHST4 | carbohydrate sulfotransferase 4-like |
| 15344585 | 6.6 | 5.38 | 2.33 | 0.0009 | TMEM61 | Transmembrane Protein 61 |
| 15338140 | 8.2 | 6.24 | 2.32 | 0.0005 | XPNPEP2 | X-prolyl aminopeptidase (aminopeptidase P) 2, membrane-bound |
| 15337135 | 6.93 | 5.72 | 2.31 | 0.0005 | EDA | ectodysplasin-A |
| 15268302 | 7.32 | 6.22 | 2.15 | 0.0030 | CCL24 | chemokine ligand 24-like protein |
| 15334332 | 5.17 | 4.07 | 2.15 | 0.0064 | SATL1 | Spermidine/Spermine N1-Acetyl Transferase-Like 1 |
| 15195879 | 7.05 | 5.99 | 2.09 | 0.0107 | RNF152 | ring finger protein 152 |
| 15183888 | 5.98 | 4.93 | 2.07 | 0.0102 | n.a. |  |
| 15240786 | 7.17 | 6.13 | 2.06 | 0.0015 | SATB2 | DNA-binding protein SATB2 |
| 15342421 | 4.52 | 3.51 | 2.01 | 0.0177 | n.a. |  |
| 15222738 | 7.32 | 8.6 | -2.01 | 0.0050 | PRSS7 | transmembrane protease, serine 15 |
| 15226312 | 4.56 | 5.61 | -2.06 | 0.0030 | PHYHIPL | phytanoyl-CoA 2-hydroxylase interacting protein |
| 15278644 | 3.28 | 4.39 | -2.15 | 0.0251 | CALB1 | calbindin 1, 28kDa |
| 15310539 | 4.09 | 5.22 | -2.19 | 0.0140 | HOMER2 | Homer Scaffolding Protein 2 |
| 15350529 | 5.29 | 6.49 | -2.31 | 0.0046 | CYP2C36 | cytochrome P450 2C36 |
| 15348027 | 5.55 | 6.79 | -2.36 | 0.0121 | n.a. |  |
| 15341561 | 6.19 | 7.19 | -2.42 | 0.0050 | TNFSF15 | Tumor Necrosis Factor (Ligand) Superfamily, Member 15 |
| 15351227 | 6.77 | 8.37 | -3.05 | 0.0120 | FOLH1 | Folate Hydrolase (Prostate-Specific Membrane Antigen) 1 |
| 15227776 | 7.59 | 9.26 | -3.17 | 0.0049 | CYP2C49 | cytochrome P450 2C49 |
