# Supplementary material for: Molecular networks affected by neonatal microbial colonization in porcine jejunum, luminally perfused with enterotoxigenic Escherichia coli, F4ac fimbria or Lactobacillus amylovorus
Source: PLoS One. 2018 Aug 30;13(8):e0202160. doi: 10.1371/journal.pone.0202160 (PMC6116929; doi:10.1371/journal.pone.0202160)
Supplement: S7 Table — NES, normalized enrichment score; FDR, false discovery rate. (DOCX) [file pone.0202160.s009.docx]

**S7 Table.** **Ordered list of the first twenty groups of genes up-regulated in CA treated pigs, compared to SA pigs (NES, normalized enrichment score; FDR, false discovery rate).**

| NAME | SIZE | NES | FDR q-val |
| --- | --- | --- | --- |
| T_CELL_ACTIVATION | 38 | 2.455 | 0.000 |
| LYMPHOCYTE_ACTIVATION | 49 | 2.366 | 0.000 |
| LEUKOCYTE_ACTIVATION | 55 | 2.301 | 0.000 |
| IMMUNE_RESPONSE | 179 | 2.299 | 0.000 |
| POSITIVE_REGULATION_OF_IMMUNE_SYSTEM_PROCESS | 41 | 2.293 | 0.000 |
| IMMUNE_SYSTEM_PROCESS | 256 | 2.266 | 0.000 |
| CELLULAR_DEFENSE_RESPONSE | 36 | 2.246 | 0.000 |
| REGULATION_OF_IMMUNE_SYSTEM_PROCESS | 52 | 2.221 | 0.000 |
| CELL_ACTIVATION | 60 | 2.175 | 0.000 |
| T_CELL_DIFFERENTIATION | 15 | 2.148 | 0.000 |
| POSITIVE_REGULATION_OF_MULTICELLULAR_ORGANISMAL_PROCESS | 54 | 2.128 | 0.001 |
| POSITIVE_REGULATION_OF_IMMUNE_RESPONSE | 24 | 2.127 | 0.001 |
| REGULATION_OF_T_CELL_ACTIVATION | 25 | 2.122 | 0.001 |
| REGULATION_OF_LYMPHOCYTE_ACTIVATION | 29 | 2.104 | 0.001 |
| REGULATION_OF_IMMUNE_RESPONSE | 28 | 2.093 | 0.001 |
| ADAPTIVE_IMMUNE_RESPONSE | 21 | 2.069 | 0.002 |
| ADAPTIVE_IMMUNE_RESPONSE_GO_0002460 | 20 | 2.050 | 0.003 |
| ACTIVATION_OF_IMMUNE_RESPONSE | 14 | 2.042 | 0.003 |
| POSITIVE_REGULATION_OF_T_CELL_ACTIVATION | 18 | 2.025 | 0.003 |
| POSITIVE_REGULATION_OF_LYMPHOCYTE_ACTIVATION | 19 | 2.015 | 0.003 |
