# Supplementary material for: Molecular networks affected by neonatal microbial colonization in porcine jejunum, luminally perfused with enterotoxigenic Escherichia coli, F4ac fimbria or Lactobacillus amylovorus
Source: PLoS One. 2018 Aug 30;13(8):e0202160. doi: 10.1371/journal.pone.0202160 (PMC6116929; doi:10.1371/journal.pone.0202160)
Supplement: S8 Table — NES, normalized enrichment score; FDR, false discovery rate. (DOCX) [file pone.0202160.s010.docx]

**S8 Table.** **Ordered list of the first twenty groups of genes up-regulated in SA treated pigs, compared to CA pigs (NES, normalized enrichment score; FDR, false discovery rate).**

| NAME | SIZE | NES | FDR q-val |
| --- | --- | --- | --- |
| ELECTRON_TRANSPORT_GO_0006118 | 43.000 | -2.064 | 0.000 |
| HETEROCYCLE_METABOLIC_PROCESS | 21.000 | -1.976 | 0.002 |
| ISOMERASE_ACTIVITY | 30.000 | -1.968 | 0.000 |
| OXIDOREDUCTASE_ACTIVITY_ACTING_ON_THE_CH_CH_GROUP_OF_DONORS | 18.000 | -1.959 | 0.000 |
| COENZYME_METABOLIC_PROCESS | 31.000 | -1.925 | 0.000 |
| FATTY_ACID_OXIDATION | 15.000 | -1.912 | 0.002 |
| COFACTOR_METABOLIC_PROCESS | 44.000 | -1.906 | 0.000 |
| AROMATIC_COMPOUND_METABOLIC_PROCESS | 23.000 | -1.867 | 0.002 |
| S_ADENOSYLMETHIONINE_DEPENDENT_METHYLTRANSFERASE_ACTIVITY | 17.000 | -1.846 | 0.000 |
| AMINO_ACID_CATABOLIC_PROCESS | 20.000 | -1.834 | 0.004 |
| AMINE_CATABOLIC_PROCESS | 20.000 | -1.815 | 0.002 |
| MITOCHONDRION | 268.000 | -1.807 | 0.000 |
| AMINO_ACID_METABOLIC_PROCESS | 65.000 | -1.797 | 0.000 |
| STEROID_BIOSYNTHETIC_PROCESS | 21.000 | -1.788 | 0.002 |
| CARBOXYLIC_ACID_METABOLIC_PROCESS | 143.000 | -1.788 | 0.000 |
| ACTIN_FILAMENT_BUNDLE_FORMATION | 12.000 | -1.780 | 0.004 |
| ACTIN_FILAMENT | 13.000 | -1.773 | 0.006 |
| ORGANIC_ACID_METABOLIC_PROCESS | 144.000 | -1.771 | 0.000 |
| ACTIN_FILAMENT_BINDING | 21.000 | -1.767 | 0.007 |
| PROTEASOME_COMPLEX | 18.000 | -1.761 | 0.004 |
