# Supplementary material for: Molecular networks affected by neonatal microbial colonization in porcine jejunum, luminally perfused with enterotoxigenic Escherichia coli, F4ac fimbria or Lactobacillus amylovorus
Source: PLoS One. 2018 Aug 30;13(8):e0202160. doi: 10.1371/journal.pone.0202160 (PMC6116929; doi:10.1371/journal.pone.0202160)
Supplement: S9 Table — (DOCX) [file pone.0202160.s011.docx]

**S9 Table 9.** **Ordered list of the 10 first statistically significant genes (false discovery rate – FDR-, P<0.05) up-regulated in CA treated pigs, compared to SA pigs.**

| Transcript Cluster ID | CA, Signal (log2) | SA, Signal (log2) | Fold Change (linear) | FDR p-value (CA vs. SA) | Gene Symbol | Description |
| --- | --- | --- | --- | --- | --- | --- |
| 15208652 | 8.82 | 8.2 | 1.53 | 1.7E-05 | CCL5 | Chemokine (C-C motif) ligand 5 |
| 15218187 | 6.39 | 5.68 | 1.63 | 2.2E-05 | CD96 | CD96 molecule |
| 15326058 | 8.48 | 7.94 | 1.45 | 8.8E-05 | CD3E | CD3e molecule, epsilon (CD3-TCR complex) |
| 15222505 | 7.62 | 7.02 | 1.52 | 1.8E-04 | TRAT1 | T-cell receptor-associated transmembrane adapter 1-like |
| 15351011 | 5.56 | 5.09 | 1.39 | 5.4E-04 | C4BPA | Complement Component 4 Binding Protein, Alpha |
| 15213398 | 6.65 | 6.17 | 1.39 | 6.9E-04 | ITGAE | Integrin, alpha E - antigen CD103 - human mucosal lymphocyte antigen 1; alpha polypeptide |
| 15286118 | 8.15 | 7.71 | 1.36 | 8.1E-04 | CD2 | CD2 molecule |
| 15205316 | 6.95 | 6.28 | 1.59 | 1.1E-03 | TBC1D4 | TBC1 domain family member 4-like |
| 15324771 | 4.97 | 4.38 | 1.51 | 1.2E-03 | ZNF215 | Zinc Finger Protein 215 |
| 15215162 | 6.25 | 5.85 | 1.32 | 1.3E-03 | CCR9 | Chemokine (C-C motif) receptor 9 |
