# Supplementary material for: Molecular networks affected by neonatal microbial colonization in porcine jejunum, luminally perfused with enterotoxigenic Escherichia coli, F4ac fimbria or Lactobacillus amylovorus
Source: PLoS One. 2018 Aug 30;13(8):e0202160. doi: 10.1371/journal.pone.0202160 (PMC6116929; doi:10.1371/journal.pone.0202160)
Supplement: S10 Table — Up-regulated gene sets present in both CA and SA pigs inside each treatment are colored in yellow. (DOCX) [file pone.0202160.s012.docx]

**S10 Table. List of the first fifty groups of genes up-regulated in differently treated loops, compared to CTRL loops and in CA or SA treated pigs.** Up-regulated gene sets present in both CA and SA pigs inside each treatment are colored in yellow.

| ETEC | | F4 | | LAB | |
| --- | --- | --- | --- | --- | --- |
| CA | SA | CA | SA | CA | SA |
| RNA_PROCESSING | RNA_PROCESSING | HETEROCYCLE_METABOLIC_PROCESS | MITOTIC_SISTER_CHROMATID_SEGREGATION | SPHINGOLIPID_METABOLIC_PROCESS | EXOPEPTIDASE_ACTIVITY |
| PROTEASOME_COMPLEX | RNA_SPLICING | PIGMENT_METABOLIC_PROCESS | SISTER_CHROMATID_SEGREGATION | EXOPEPTIDASE_ACTIVITY | M_PHASE_OF_MITOTIC_CELL_CYCLE |
| RNA_SPLICING | PROTEASOME_COMPLEX | PHOSPHORIC_DIESTER_HYDROLASE_ACTIVITY | MITOSIS |  | M_PHASE |
| RESPONSE_TO_BIOTIC_STIMULUS | INFLAMMATORY_RESPONSE | SODIUM_ION_TRANSPORT | CHEMOKINE_RECEPTOR_BINDING | | MITOSIS |
| CHEMOKINE_RECEPTOR_BINDING | CHEMOKINE_ACTIVITY | PHOSPHORIC_ESTER_HYDROLASE_ACTIVITY | G_PROTEIN_COUPLED_RECEPTOR_BINDING | |  |
| CHEMOKINE_ACTIVITY | JAK_STAT_CASCADE | ANION_TRANSPORT | SPHINGOLIPID_METABOLIC_PROCESS |  |  |
| RNA_SPLICING_FACTOR_ACTIVITYTRANSESTERIFICATION_MECHANISM | CHEMOKINE_RECEPTOR_BINDING | TRANSFERASE_ACTIVITY_TRANSFERRING_SULFUR_CONTAINING_GROUPS | M_PHASE_OF_MITOTIC_CELL_CYCLE | |  |
| MRNA_PROCESSING_GO_0006397 | DEFENSE_RESPONSE | PIGMENT_BIOSYNTHETIC_PROCESS |  |  |  |
| RIBONUCLEOPROTEIN_COMPLEX | CYTOKINE_BINDING | EXOPEPTIDASE_ACTIVITY |  |  |  |
| SMALL_NUCLEAR_RIBONUCLEOPROTEIN_COMPLEX | RESPONSE_TO_WOUNDING | SPHINGOLIPID_METABOLIC_PROCESS |  |  |  |
| G_PROTEIN_COUPLED_RECEPTOR_BINDING | CYTOKINE_ACTIVITY |  |  |  |  |
| LOCOMOTORY_BEHAVIOR | LOCOMOTORY_BEHAVIOR |  |  |  |  |
| PHOSPHOLIPASE_C_ACTIVITY | RIBONUCLEOPROTEIN_COMPLEX |  |  |  |  |
| ENDOPLASMIC_RETICULUM_PART | G_PROTEIN_COUPLED_RECEPTOR_BINDING |  |  |  |  |
| PEPTIDYL_AMINO_ACID_MODIFICATION | MRNA_PROCESSING_GO_0006397 |  |  |  |  |
| ENDOPLASMIC_RETICULUM_LUMEN | MITOSIS |  |  |  |  |
| RNA_SPLICINGVIA_TRANSESTERIFICATION_REACTIONS | M_PHASE_OF_MITOTIC_CELL_CYCLE | |  |  |  |
| DNA_INTEGRITY_CHECKPOINT | M_PHASE |  |  |  |  |
| JAK_STAT_CASCADE | IMMUNE_RESPONSE |  |  |  |  |
| CYTOKINE_AND_CHEMOKINE_MEDIATED_SIGNALING_PATHWAY | NUCLEOTIDE_METABOLIC_PROCESS |  |  |  |  |
| IMMUNE_RESPONSE | RESPONSE_TO_EXTERNAL_STIMULUS | |  |  |  |
| PROTEIN_FOLDING | MITOTIC_CELL_CYCLE |  |  |  |  |
| RESPONSE_TO_OTHER_ORGANISM | RRNA_METABOLIC_PROCESS |  |  |  |  |
| INTRINSIC_TO_ENDOPLASMIC_RETICULUM_MEMBRANE | CELL_CYCLE_PHASE |  |  |  |  |
| DEFENSE_RESPONSE | NUCLEOBASENUCLEOSIDE_AND_NUCLEOTIDE_METABOLIC_PROCESS | | |  |  |
| INTEGRAL_TO_ENDOPLASMIC_RETICULUM_MEMBRANE | CELL_CYCLE_PROCESS |  |  |  |  |
| MRNA_METABOLIC_PROCESS | UNFOLDED_PROTEIN_BINDING |  |  |  |  |
| ENDOPLASMIC_RETICULUM | CHROMOSOME |  |  |  |  |
| DNA_RECOMBINATION | BEHAVIOR |  |  |  |  |
| REGULATION_OF_RESPONSE_TO_STIMULUS | NUCLEOLUS |  |  |  |  |
| RIBONUCLEASE_ACTIVITY | CHROMOSOMAL_PART |  |  |  |  |
| CYTOKINE_ACTIVITY | PEPTIDYL_TYROSINE_PHOSPHORYLATION | |  |  |  |
| VESICLE_MEMBRANE | INTERLEUKIN_BINDING |  |  |  |  |
| RIBOSOME | NUCLEAR_CHROMOSOME |  |  |  |  |
| ADAPTIVE_IMMUNE_RESPONSE_GO_0002460 | MRNA_METABOLIC_PROCESS |  |  |  |  |
| RESPONSE_TO_VIRUS | REPLICATION_FORK |  |  |  |  |
| CYTOKINE_BINDING | REGULATION_OF_MITOSIS |  |  |  |  |
| HEMATOPOIETIN_INTERFERON_CLASSD200_DOMAIN_CYTOKINE_RECEPTOR_BINDING | REGULATION_OF_PROTEIN_AMINO_ACID_PHOSPHORYLATION | |  |  |  |
| REPLICATION_FORK | TYROSINE_PHOSPHORYLATION_OF_STAT_PROTEIN |  |  |  |  |
| GENERAL_RNA_POLYMERASE_II_TRANSCRIPTION_FACTOR_ACTIVITY | RNA_HELICASE_ACTIVITY |  |  |  |  |
| CHROMOSOMAL_PART | SUGAR_BINDING |  |  |  |  |
| TRNA_METABOLIC_PROCESS | PEPTIDYL_TYROSINE_MODIFICATION |  |  |  |  |
| REGULATION_OF_CYCLIN_DEPENDENT_PROTEIN_KINASE_ACTIVITY | NUCLEAR_PART |  |  |  |  |
| CYTOPLASMIC_VESICLE_MEMBRANE | IMMUNE_SYSTEM_PROCESS |  |  |  |  |
| CYTOPLASMIC_VESICLE_PART | RNA_SPLICINGVIA_TRANSESTERIFICATION_REACTIONS |  |  |  |  |
| ENDOPLASMIC_RETICULUM_MEMBRANE | REGULATION_OF_PEPTIDYL_TYROSINE_PHOSPHORYLATION |  |  |  |  |
| TRANSFERASE_ACTIVITY_TRANSFERRING_ALKYL_OR_ARYLOTHER_THAN_METHYLGROUPS | ATP_DEPENDENT_RNA_HELICASE_ACTIVITY | |  |  |  |
| GUANYL_NUCLEOTIDE_BINDING | RNA_BINDING |  |  |  |  |
| ER_GOLGI_INTERMEDIATE_COMPARTMENT | PROTEIN_KINASE_CASCADE |  |  |  |  |
| ADAPTIVE_IMMUNE_RESPONSE | RNA_DEPENDENT_ATPASE_ACTIVITY | |  |  |  |
