# Supplementary material for: Molecular networks affected by neonatal microbial colonization in porcine jejunum, luminally perfused with enterotoxigenic Escherichia coli, F4ac fimbria or Lactobacillus amylovorus
Source: PLoS One. 2018 Aug 30;13(8):e0202160. doi: 10.1371/journal.pone.0202160 (PMC6116929; doi:10.1371/journal.pone.0202160)
Supplement: S11 Table — Down-regulated gene sets present in both CA and SA pigs inside each treatment are colored in yellow. (DOCX) [file pone.0202160.s013.docx]

**S11 Table.** **List of the first fifty groups of genes down-regulated in differently treated loops, compared to CTRL loops and in CA or SA treated pigs.** Down-regulated gene sets present in both CA and SA pigs inside each treatment are colored in yellow.

| ETEC | | F4 | | LAB | |
| --- | --- | --- | --- | --- | --- |
| CA | SA | CA | SA | CA | SA |
| SPHINGOLIPID_METABOLIC_PROCESS | LIPID_TRANSPORT | TRANSLATION_REGULATOR_ACTIVITY | RIBONUCLEOPROTEIN_COMPLEX_BIOGENESIS_AND_ASSEMBLY | STRUCTURAL_CONSTITUENT_OF_RIBOSOME | STRUCTURAL_CONSTITUENT_OF_RIBOSOME |
| ORGANIC_ACID_METABOLIC_PROCESS | SPHINGOLIPID_METABOLIC_PROCESS | TRANSLATION_FACTOR_ACTIVITY_NUCLEIC_ACID_BINDING | TRANSLATIONAL_INITIATION | RIBONUCLEOPROTEIN_COMPLEX_BIOGENESIS_AND_ASSEMBLY | RIBONUCLEOPROTEIN_COMPLEX_BIOGENESIS_AND_ASSEMBLY |
| SECONDARY_ACTIVE_TRANSMEMBRANE_TRANSPORTER_ACTIVITY | LIGAND_DEPENDENT_NUCLEAR_RECEPTOR_ACTIVITY | RIBOSOME_BIOGENESIS_AND_ASSEMBLY | PROTEIN_RNA_COMPLEX_ASSEMBLY | RIBOSOME_BIOGENESIS_AND_ASSEMBLY | CELLULAR_BIOSYNTHETIC_PROCESS |
| CARBOXYLIC_ACID_METABOLIC_PROCESS | SECONDARY_ACTIVE_TRANSMEMBRANE_TRANSPORTER_ACTIVITY | RIBONUCLEOPROTEIN_COMPLEX_BIOGENESIS_AND_ASSEMBLY | TRANSLATION_REGULATOR_ACTIVITY | TRANSLATION | RIBOSOME_BIOGENESIS_AND_ASSEMBLY |
| LIGAND_DEPENDENT_NUCLEAR_RECEPTOR_ACTIVITY | RHO_PROTEIN_SIGNAL_TRANSDUCTION | RNA_PROCESSING | TRANSLATION_FACTOR_ACTIVITY_NUCLEIC_ACID_BINDING | TRNA_METABOLIC_PROCESS | TRANSLATIONAL_INITIATION |
| CELLULAR_LIPID_METABOLIC_PROCESS | SYMPORTER_ACTIVITY | TRANSLATIONAL_INITIATION | STRUCTURAL_CONSTITUENT_OF_RIBOSOME | RNA_PROCESSING | TRANSLATION |
| OXIDOREDUCTASE_ACTIVITY_ACTING_ON_THE_CH_CH_GROUP_OF_DONORS | CARBOXYLIC_ACID_METABOLIC_PROCESS | IMMUNE_RESPONSE | TRANSLATION_INITIATION_FACTOR_ACTIVITY |  | CALMODULIN_BINDING |
| AMINO_ACID_AND_DERIVATIVE_METABOLIC_PROCESS | EXTRACELLULAR_MATRIX | NUCLEOLUS | CALMODULIN_BINDING |  | TRANSLATION_INITIATION_FACTOR_ACTIVITY |
| EPIDERMAL_GROWTH_FACTOR_RECEPTOR_SIGNALING_PATHWAY | ORGANIC_ACID_TRANSPORT | TRANSLATION_INITIATION_FACTOR_ACTIVITY | CELLULAR_BIOSYNTHETIC_PROCESS |  | REGULATION_OF_CELLULAR_COMPONENT_ORGANIZATION_AND_BIOGENESIS |
| OXIDOREDUCTASE_ACTIVITY | AMINE_TRANSPORT | REGULATION_OF_CELLULAR_PROTEIN_METABOLIC_PROCESS | BLOOD_COAGULATION | | REGULATION_OF_NEUROTRANSMITTER_LEVELS |
| LIPID_TRANSPORT | CDC42_PROTEIN_SIGNAL_TRANSDUCTION | TRNA_METABOLIC_PROCESS | NEGATIVE_REGULATION_OF_PROTEIN_METABOLIC_PROCESS |  |  |
| LIPID_METABOLIC_PROCESS | AMINO_ACID_AND_DERIVATIVE_METABOLIC_PROCESS | REGULATION_OF_TRANSLATIONAL_INITIATION | HEMOSTASIS | |  |
| NITROGEN_COMPOUND_METABOLIC_PROCESS | PROTEINACEOUS_EXTRACELLULAR_MATRIX | RRNA_METABOLIC_PROCESS | NEGATIVE_REGULATION_OF_CELLULAR_PROTEIN_METABOLIC_PROCESS |  |  |
| COFACTOR_BINDING | ORGANIC_ACID_METABOLIC_PROCESS | RIBONUCLEOPROTEIN_COMPLEX | COAGULATION | |  |
| OXIDOREDUCTASE_ACTIVITY_ACTING_ON_THE_ALDEHYDE_OR_OXO_GROUP_OF_DONORS | CARBOXYLIC_ACID_TRANSPORT | TRANSLATION | TRANSLATION |  |  |
| FATTY_ACID_METABOLIC_PROCESS | ACTIVE_TRANSMEMBRANE_TRANSPORTER_ACTIVITY | REGULATION_OF_PROTEIN_MODIFICATION_PROCESS | REGULATION_OF_TRANSLATIONAL_INITIATION |  |  |
| AMINE_METABOLIC_PROCESS | AMINO_ACID_TRANSPORT | POSITIVE_REGULATION_OF_IMMUNE_RESPONSE | RIBOSOME_BIOGENESIS_AND_ASSEMBLY |  |  |
| PEROXISOME | MICROBODY | REGULATION_OF_PEPTIDYL_TYROSINE_PHOSPHORYLATION | NUCLEOLAR_PART | |  |
| COENZYME_BINDING | AMINE_CATABOLIC_PROCESS | RNA_HELICASE_ACTIVITY | NEGATIVE_REGULATION_OF_BIOSYNTHETIC_PROCESS |  |  |
| MICROBODY | PEROXISOME | CELLULAR_COMPONENT_DISASSEMBLY | NEGATIVE_REGULATION_OF_TRANSLATION | | |
| LIPID_CATABOLIC_PROCESS | LIPID_METABOLIC_PROCESS | NLS_BEARING_SUBSTRATE_IMPORT_INTO_NUCLEUS | CYTOKINE_SECRETION | | |
| ELECTRON_CARRIER_ACTIVITY | AMINO_ACID_CATABOLIC_PROCESS | REGULATION_OF_PROTEIN_METABOLIC_PROCESS | NEGATIVE_REGULATION_OF_CELLULAR_BIOSYNTHETIC_PROCESS | | |
| AMINO_ACID_METABOLIC_PROCESS | AMINO_ACID_METABOLIC_PROCESS | REGULATION_OF_PROTEIN_AMINO_ACID_PHOSPHORYLATION | GLUCOSE_METABOLIC_PROCESS | | |
| SYMPORTER_ACTIVITY | NITROGEN_COMPOUND_CATABOLIC_PROCESS | POSITIVE_REGULATION_OF_IMMUNE_SYSTEM_PROCESS | CALCIUM_ION_BINDING | | |
| EXCRETION | AMINO_ACID_DERIVATIVE_METABOLIC_PROCESS | INOSITOL_OR_PHOSPHATIDYLINOSITOL_KINASE_ACTIVITY | REGULATION_OF_BODY_FLUID_LEVELS | | |
| CELLULAR_LIPID_CATABOLIC_PROCESS | NITROGEN_COMPOUND_METABOLIC_PROCESS | HELICASE_ACTIVITY | REGULATION_OF_CELLULAR_PROTEIN_METABOLIC_PROCESS |  |  |
| AMINO_ACID_TRANSPORT | COENZYME_BINDING | REGULATION_OF_CELLULAR_COMPONENT_ORGANIZATION_AND_BIOGENESIS | OXIDOREDUCTASE_ACTIVITY_GO_0016616 | | |
| MEMBRANE_LIPID_METABOLIC_PROCESS | CELLULAR_LIPID_METABOLIC_PROCESS | POSITIVE_REGULATION_OF_PHOSPHATE_METABOLIC_PROCESS | WOUND_HEALING | |  |
| MITOCHONDRION | AMINE_METABOLIC_PROCESS | NEGATIVE_REGULATION_OF_CELLULAR_PROTEIN_METABOLIC_PROCESS | REGULATION_OF_CELLULAR_COMPONENT_ORGANIZATION_AND_BIOGENESIS |  |  |
| GENERATION_OF_PRECURSOR_METABOLITES_AND_ENERGY | REGULATION_OF_NEUROTRANSMITTER_LEVELS | ACTIVATION_OF_IMMUNE_RESPONSE | GENERATION_OF_PRECURSOR_METABOLITES_AND_ENERGY | | |
| ACTIVE_TRANSMEMBRANE_TRANSPORTER_ACTIVITY | EXTRACELLULAR_MATRIX_PART | POSITIVE_REGULATION_OF_PHOSPHORYLATION | REGULATION_OF_RESPONSE_TO_EXTERNAL_STIMULUS | | |
| TRANSMEMBRANE_RECEPTOR_PROTEIN_TYROSINE_KINASE_SIGNALING_PATHWAY | COFACTOR_BINDING | POSITIVE_REGULATION_OF_RESPONSE_TO_STIMULUS | REGULATION_OF_TRANSLATION |  |  |
| MONOCARBOXYLIC_ACID_METABOLIC_PROCESS | L_AMINO_ACID_TRANSMEMBRANE_TRANSPORTER_ACTIVITY | PROTEIN_RNA_COMPLEX_ASSEMBLY | REGULATION_OF_PEPTIDYL_TYROSINE_PHOSPHORYLATION |  |  |
| SECONDARY_METABOLIC_PROCESS | DIGESTION | NEGATIVE_REGULATION_OF_PROTEIN_METABOLIC_PROCESS | OXIDOREDUCTASE_ACTIVITY_ACTING_ON_CH_OH_GROUP_OF_DONORS | | |
| CARBOHYDRATE_METABOLIC_PROCESS | EXOPEPTIDASE_ACTIVITY | UNFOLDED_PROTEIN_BINDING | TRNA_METABOLIC_PROCESS |  |  |
| AMINE_TRANSPORT | LIPID_CATABOLIC_PROCESS | PROTEIN_FOLDING | CARBOHYDRATE_KINASE_ACTIVITY |  |  |
| ENZYME_LINKED_RECEPTOR_PROTEIN_SIGNALING_PATHWAY | REGULATION_OF_ANATOMICAL_STRUCTURE_MORPHOGENESIS | PEPTIDYL_TYROSINE_MODIFICATION | LEUKOCYTE_MIGRATION | | |
| HYDRO_LYASE_ACTIVITY | OXIDOREDUCTASE_ACTIVITY | REGULATION_OF_IMMUNE_RESPONSE | BIOSYNTHETIC_PROCESS | | |
| PHOSPHATE_TRANSMEMBRANE_TRANSPORTER_ACTIVITY | EXCRETION | REGULATION_OF_TRANSLATION | PROTEIN_SECRETION | | |
| NITROGEN_COMPOUND_BIOSYNTHETIC_PROCESS | INORGANIC_ANION_TRANSMEMBRANE_TRANSPORTER_ACTIVITY | JAK_STAT_CASCADE | PHOSPHOLIPASE_C_ACTIVATION | | |
| GLAND_DEVELOPMENT | REGULATION_OF_RHO_PROTEIN_SIGNAL_TRANSDUCTION | LYMPHOCYTE_ACTIVATION | RESPONSE_TO_ORGANIC_SUBSTANCE | | |
| CARBOXYLIC_ACID_TRANSPORT | REGULATION_OF_RAS_PROTEIN_SIGNAL_TRANSDUCTION | REGULATION_OF_IMMUNE_SYSTEM_PROCESS | LIGASE_ACTIVITY_FORMING_CARBON_OXYGEN_BONDS |  |  |
| ORGANIC_ACID_TRANSPORT | REGULATION_OF_SMALL_GTPASE_MEDIATED_SIGNAL_TRANSDUCTION | NEGATIVE_REGULATION_OF_CELLULAR_COMPONENT_ORGANIZATION_AND_BIOGENESIS | RHYTHMIC_PROCESS | | |
| AMINO_ACID_DERIVATIVE_METABOLIC_PROCESS | NEURITE_DEVELOPMENT | RNA_DEPENDENT_ATPASE_ACTIVITY | NUCLEOLUS |  |  |
| INORGANIC_ANION_TRANSMEMBRANE_TRANSPORTER_ACTIVITY | PHOSPHATE_TRANSMEMBRANE_TRANSPORTER_ACTIVITY | PROTEIN_COMPLEX_DISASSEMBLY | REGULATION_OF_SECRETION | | |
| ACTIN_FILAMENT_BUNDLE_FORMATION | OXIDOREDUCTASE_ACTIVITY_ACTING_ON_THE_CH_CH_GROUP_OF_DONORS | RAS_GTPASE_ACTIVATOR_ACTIVITY | MACROMOLECULE_BIOSYNTHETIC_PROCESS |  |  |
| RHO_PROTEIN_SIGNAL_TRANSDUCTION | HYDROGEN_ION_TRANSMEMBRANE_TRANSPORTER_ACTIVITY | GTPASE_REGULATOR_ACTIVITY | RRNA_METABOLIC_PROCESS |  |  |
| ANION_CATION_SYMPORTER_ACTIVITY | REGULATION_OF_GTPASE_ACTIVITY | POSITIVE_REGULATION_OF_CELLULAR_PROTEIN_METABOLIC_PROCESS | RESPONSE_TO_WOUNDING |  |  |
| MEMBRANE_FRACTION | AMINE_TRANSMEMBRANE_TRANSPORTER_ACTIVITY | IMMUNE_SYSTEM_PROCESS | REGULATION_OF_PROTEIN_METABOLIC_PROCESS |  |  |
| LIPID_TRANSPORTER_ACTIVITY | OXIDOREDUCTASE_ACTIVITY_ACTING_ON_CH_OH_GROUP_OF_DONORS | MRNA_BINDING | JAK_STAT_CASCADE |  |  |
